# CLASP2 facilitates dynamic actin filament organization along the microtubule lattice

**DOI:** 10.1091/mbc.E22-05-0149

**Published:** 2023-02-21

**Authors:** N. C. Rodgers, E. J. Lawrence, A. V. Sawant, N. Efimova, G. Gonzalez-Vasquez, T. T. Hickman, I. Kaverina, M. Zanic

**Affiliations:** a Chemical and Physical Biology Graduate Program, Vanderbilt University, Nashville, TN 37232; b Department of Cell and Development Biology, Vanderbilt University, Nashville, TN 37232; c Interdisciplinary Graduate Program, Vanderbilt University, Nashville, TN 37232; d Quantitative and Chemical Biology Graduate Program, Vanderbilt University, Nashville, TN 37232; e Department of Chemical and Biomolecular Engineering, Vanderbilt University, Nashville, TN 37232; f Department of Biochemistry, Vanderbilt University, Nashville, TN 37232; CEA Grenoble

## Abstract

Coordination between the microtubule and actin networks is essential for cell motility, neuronal growth cone guidance, and wound healing. Members of the CLASP (cytoplasmic linker–associated protein) family of proteins have been implicated in the cytoskeletal cross-talk between microtubules and actin networks; however, the molecular mechanisms underlying the role of CLASP in cytoskeletal coordination are unclear. Here, we investigate CLASP2α’s cross-linking function with microtubules and F-actin. Our results demonstrate that CLASP2α cross-links F-actin to the microtubule lattice in vitro. We find that the cross-linking ability is retained by L-TOG2-S, a minimal construct containing the TOG2 domain and serine-arginine–rich region of CLASP2α. Furthermore, CLASP2α promotes the accumulation of multiple actin filaments along the microtubule, supporting up to 11 F-actin landing events on a single microtubule lattice region. CLASP2α also facilitates the dynamic organization of polymerizing actin filaments templated by the microtubule network, with F-actin forming bridges between individual microtubules. Finally, we find that depletion of CLASPs in vascular smooth muscle cells results in disorganized actin fibers and reduced coalignment of actin fibers with microtubules, suggesting that CLASP and microtubules contribute to higher-order actin structures. Taken together, our results indicate that CLASP2α can directly cross-link F-actin to microtubules and that this microtubule-CLASP-actin interaction may influence overall cytoskeletal organization in cells.

## INTRODUCTION

The cytoskeleton is an essential cellular component that drives a multitude of processes such as cell division and motility and defines cell shape. Individual components of the cytoskeleton must coordinate to perform their cellular functions (Rodriguez *et al.*, 2003; Dogterom and Koenderink, 2019; Pimm and Henty-Ridilla, 2021). For example, microtubules and actin interact with each other to facilitate cell motility (Waterman-Storer and Salmon, 1997, 1999; Ballestrem *et al.*, 2000; Salmon *et al.*, 2002 Wu *et al.*, 2008) and growth cone guidance (Rochlin *et al.*, 1999; Dent and Kalil, 2001; Schaefer *et al.*, 2002; Slater *et al.*, 2019). Many cellular factors are involved in this coordination (Rodriguez *et al.*, 2003), and the proteins that physically couple the microtubule and actin networks are of particular interest (Dogterom and Koenderink, 2019; Pimm and Henty-Ridilla, 2021). However, the specific interactions underlying cytoskeletal coupling remain to be fully understood.

Cytoplasmic linker–associated proteins (CLASPs) are a well-studied family of microtubule-associated proteins (MAPs) that have been implicated in interacting with both microtubules and F-actin (Tsvetkov *et al.*, 2007; Engel *et al.*, 2014; Dogterom and Koenderink, 2019). CLASPs are known as microtubule stabilizers with essential roles in cell division, cell migration, and neuronal development (Lawrence *et al.*, 2020). There are two paralogues of CLASP, CLASP1 and CLASP2, that are alternatively spliced, resulting in multiple isoforms that are differentially expressed and may have some isoform-specific functions (Akhmanova *et al.*, 2001; Lawrence *et al.*, 2020). In vitro studies with purified proteins established that CLASPs promote sustained microtubule growth by suppressing microtubule catastrophe, the transition from growth to shrinkage, while promoting microtubule rescue, the transition from shrinkage to growth (Akhmanova *et al.*, 2001; Al-Bassam *et al.*, 2010; Patel *et al.*, 2012; Leano *et al.*, 2013; Maki *et al.*, 2015; Aher *et al.*, 2018; Lawrence *et al.*, 2018). In cells, CLASPs are targeted to growing microtubule plus ends via a direct interaction with microtubule end-binding EB proteins and can specifically regulate microtubule dynamics at the actin-rich cell cortex (Mimori-Kiyosue *et al.*, 2005). Furthermore, CLASPs are involved in the process of microtubule plus-end capture at the cell cortex through interactions with LL5β, a component of the cortical microtubule stabilization complex (Lansbergen *et al.*, 2006; Hotta *et al.*, 2010; Stehbens *et al.*, 2014). CLASPs’ stabilization and anchoring of microtubules is also important for the regulation and dynamics of podosomes, polymerizing actin-based structures, in vascular smooth muscle cells (Efimova *et al.*, 2014; Zhu *et al.*, 2016). All these studies suggest CLASPs’ roles in the interplay between the microtubule and actin networks. However, it remains unclear whether CLASPs directly interact with the actin network in these contexts.

A previous study investigating CLASP-actin interaction reported that the two CLASP paralogues, CLASP1 and CLASP2, colocalize with actin stress fibers in primary fibroblasts and spinal cord neurons (Tsvetkov *et al.*, 2007). The authors found that both CLASP paralogues coimmunoprecipitated with actin from fibroblast cells and suggested that the tumor-overexpressing gene (TOG)-1 domain and serine-arginine–rich region of CLASP2α facilitate the F-actin interaction. Another study, using cosedimentation experiments with purified proteins, reported a direct interaction between F-actin and CLASP2α, as well as cosedimentation of G-actin with microtubules in the presence of CLASP2α (Engel *et al.*, 2014). To our knowledge, these two reports provide the only evidence of CLASP-actin interaction, leaving open the question of whether CLASP alone is sufficient for cross-linking of microtubules and actin filaments, which may play a role in the organization of stress fibers in cells.

## RESULTS AND DISCUSSION

### Human CLASP2α directly cross-links actin filaments to microtubules

**FIGURE 1:**
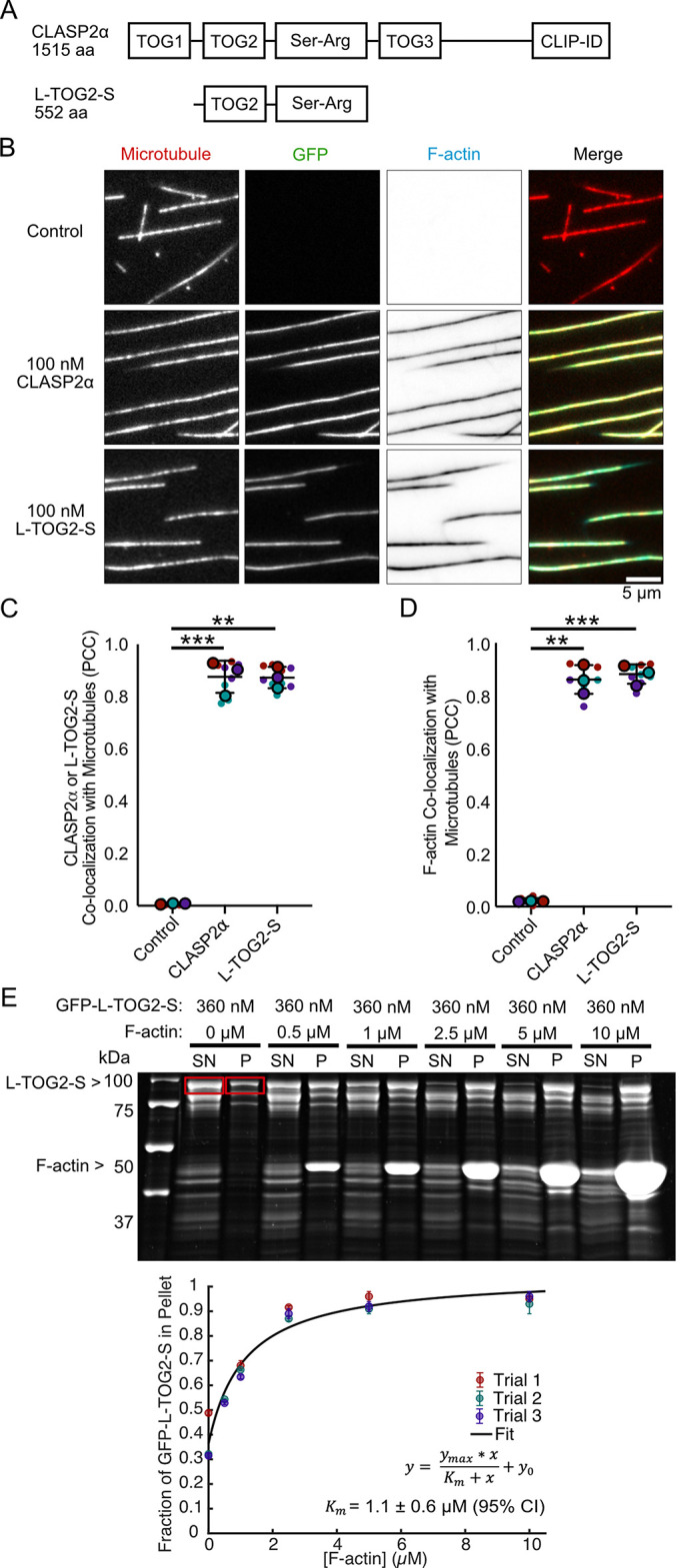
Human CLASP2α directly cross-links actin filaments to microtubules. (A) Schematic of the domain structure of human CLASP2α and minimal construct L-TOG2-S. (B) Representative images of Taxol-stabilized microtubules incubated for 10 min with storage buffer (top row), 100 nM CLASP2α-GFP (middle row), or 100 nM GFP-L-TOG2-S (bottom row) and 6.5 µM TRITC-phalloidin F-actin. (C) Quantification of colocalization between either CLASP2α or L-TOG2-S with microtubules using the PCC. (D) Quantification of colocalization between actin filaments and microtubules, using the PCC. Small data points are individual PCC measurements, and large data points are the mean PCC for three independent experimental days, represented by different colors. Error bars are the SD. Kruskal–Wallis test multiple comparisons, ** *p* < 0.01 and *** *p* < 0.001. Comparisons between CLASP2α and L-TOG2-S are ns, *p* > 0.05. (E) Example SDS–PAGE gel from the high-speed cosedimentation experiment and the quantification of the fraction of GFP-L-TOG2-S in the pellet as a function of F-actin concentration. Red boxes represent L-TOG2-S bands quantified for supernatant and pellet. Error bars represent error propagation of standard deviations. Black line is the Michaelis–Menten fit. Experiments performed in triplicate.

To investigate CLASP2α’s ability to directly cross-link microtubules and actin, we expressed and purified full-length CLASP2α-GFP using an Sf9-insect-cell–based system (Figure 1A and Supplemental Figure S1A). We employed an established in vitro reconstitution assay (Gell *et al.*, 2010) using three-color total internal reflection fluorescence microscopy (TIRF) to directly visualize microtubules, actin, and CLASP. First, we bound Alexa 647–labeled, Taxol-stabilized microtubules to the surface of a coverslip. Next, we added 6.5 µM TRITC-phalloidin–stabilized F-actin in the absence (Figure 1B, top) or presence (Figure 1B, middle) of 100 nM purified CLASP2α-GFP to the flow cell. As expected, we observed specific CLASP2α-GFP localization along the micro­tubule lattice (Figure 1B, middle). We quantified the correlation between the CLASP2α-GFP and microtubule signals after 10 min of incubation using the Pearson correlation coefficient (PCC) and measured a high degree of correlation (PCC = 0.88 ± 0.06, SD, *N* = 9 fields of view [FOVs] over three independent experimental days) (Figure 1C). The investigation of TRITC-phalloidin F-actin showed that F-actin robustly colocalized with microtubules in the presence of CLASP2α-GFP (PCC = 0.86 ± 0.05, SD, *N* = 9 FOVs over three independent experimental days) (Figure 1, B and D). No significant colocalization between microtubules and F-actin was observed in the absence of CLASP2α (PCC = 0.022 ± 0.001, SD, *N* = 9 FOVs over three independent experimental days) (Figure 1, B and D). Furthermore, we confirmed that the colocalization of F-actin with microtubules did not depend on stabilization of F-actin using phalloidin, as F-actin also localized to CLASP2-coated microtubules in the absence of phalloidin (Supplemental Figure S1B). Based on these results, we conclude that CLASP2α directly cross-links F-actin to microtubules.

Previous reports using coimmunoprecipitation from fibroblast cells implicated a serine-arginine–rich region of CLASP2α in its interaction with actin (Tsvetkov *et al.*, 2007). Recently, a minimal CLASP2 construct, L-TOG2-S, containing a single TOG2 domain and the serine-arginine–rich region (Figure 1A), was reported to recapitulate CLASP’s effect on microtubule dynamics (Aher *et al.*, 2018). We wondered whether this minimal CLASP2 construct is also sufficient to cross-link microtubules and F-actin. We used the same TIRF-based approach to investigate TRITC-phalloidin F-actin localization on microtubules in the presence of purified 100 nM GFP-L-TOG2-S (Supplemental Figure S1C). As expected, GFP-L-TOG2-S showed strong localization to microtubules (PCC = 0.87 ± 0.04, SD, *N* = 9 FOVs over three independent experimental days) (Figure 1, B, bottom, and C). Furthermore, we found that F-actin also robustly colocalized with microtubules in the presence of GFP-L-TOG2-S (PCC = 0.88 ± 0.04, SD, *N* = 9 FOVs over three independent experimental days) (Figure 1, B, bottom, and D). To further characterize the direct interaction of the L-TOG2-S minimal construct of CLASP2 with F-actin, we performed cosedimentation experiments. We measured the fraction of GFP-L-TOG2-S protein pelleting with F-actin over a range of F-actin concentrations and determined the binding affinity of the L-TOG2-S – F-actin interaction to be 1.1 ± 0.6 µM in the absence of microtubules (Figure 1E). Taken together, these results demonstrate that, in spite of the relatively weak direct interaction with F-actin, the minimal CLASP2α construct containing a single TOG2 domain and the serine-arginine–rich region is sufficient to cross-link F-actin to microtubules.

### CLASP2α mediates sequential binding of actin filaments along the microtubule lattice

**FIGURE 2:**
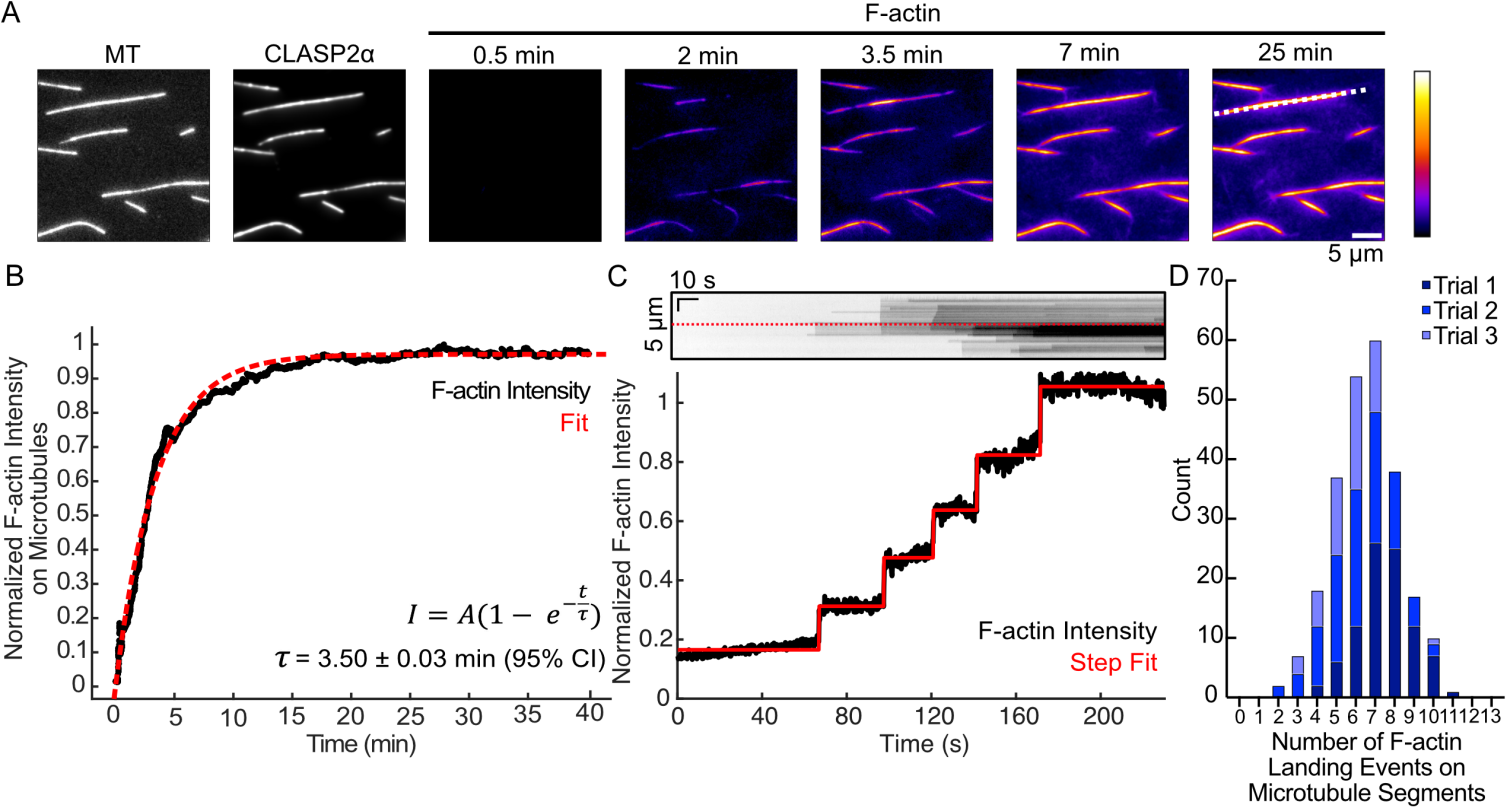
CLASP2 mediates sequential binding of actin filaments along the microtubule lattice. (A) Example time-lapse TIRF images of Taxol-stabilized microtubules in the presence of 100 nM CLASP2α-GFP and 6.5 µM TRITC-phalloidin–stabilized F-actin. White dotted line corresponds to the kymograph in panel C. (B) Example normalized actin filament intensity in microtubule areas over time for the images in panel A. Red dotted line represents the fit to the intensity-over-time equation. (C) Top, Kymograph of the F-actin channel for an example microtubule (denoted by a dotted white line in panel A) for the first 5 min. Red dotted line corresponds to the intensity line scan. Bottom: 3-pixel-wide intensity line scan for the F-actin channel in the kymograph. Red line represents the stepping algorithm fit to count steps of F-actin landing events (see *Materials and Methods*). (D) Stacked histogram of the total number of F-actin landing events on microtubule segments. Experiments done in triplicate (*N* = 91 [Trial 1], 99 [Trial 2], and 54 [Trial 3] microtubule regions analyzed).

To further investigate how actin filaments interact with microtubules in the presence of CLASP2α, we imaged TRITC-phalloidin F-actin on microtubules over time for up to 40 min. We observed the initial landing of actin filaments within 2 min (Figure 2A; Supplemental Video 1). Notably, the F-actin signal continued to increase over time, reaching saturation within the duration of the experiment (Figure 2B). A closer inspection of the increasing TRITC-phalloidin F-actin intensity on individual microtubules revealed step-like increases in fluorescence intensity on a region of microtubule lattice (Figure 2C), suggesting sequential binding of actin filaments. Using a stepping analysis algorithm (Bronson *et al.*, 2009; see *Materials and Methods*), we measured the accumulation of F-actin within a 3-pixel-wide (480 nm) microtubule segment over a period of 40 min. The mean number of landing events was 6.3 ± 0.6 (SD, *N* = 91, 99, and 54 microtubule lattice segments analyzed on three experimental days), with up to 11 sequential F-actin landing events occurring on a single microtubule segment (Figure 2D).

**Figure:** Movie S1 F-actin landing on CLASP2-coated microtubules. Time-lapse of 6.5 μM F-actin and three-color merged time-lapse. F-actin time-lapse is merged with still microtubule image and average projection of CLASP2α -GFP signal. Video playback is 300 frames per second.

We wondered whether the sequential accumulation of F-actin on microtubules could be supported by potential F-actin bundling activity of CLASP2α. To test whether CLASP2α on its own has the ability to bundle F-actin, we incubated 200 nM CLASP2α-GFP with 1 µM TRITC-phalloidin F-actin for 10 min at room temperature and then imaged using our in vitro reconstitution approach (see *Materials and Methods*). Our results showed that CLASP2α on its own does not induce F-actin bundling in these conditions (Supplemental Figure S1D). In contrast, robust F-actin bundles were observed in the presence of 200 nM fascin, a well-known actin bundler. Additionally, as expected, CLASP2α alone was sufficient to induce bundling of microtubules in these conditions. These results suggest that the CLASP2α-mediated F-actin accumulation on microtubules does not occur through bundling of actin filaments by CLASP2α.

### CLASP2α facilitates dynamic actin filament organization templated by the microtubule arrangement

**FIGURE 3:**
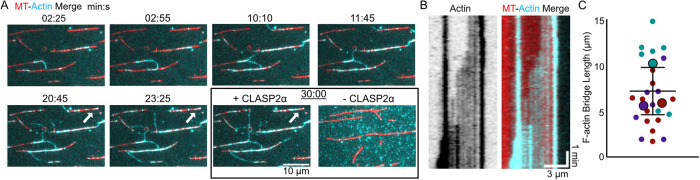
CLASP2 facilitates dynamic actin filament organization templated by the microtubule network. (A) Example time-lapse images demonstrating dynamic F-actin (cyan) connecting multiple microtubules (red). White arrow highlights an example of growing F-actin shown by a kymograph in panel B. The last image in the sequence represents the CLASP2 storage buffer control after 30 min. (B) Example kymograph demonstrating growing F-actin along a microtubule lattice (left: actin channel alone, right: merged image). (C) Quantification of the individual lengths of F-actin connections between microtubules. Smaller data points are the individual measurements, and larger data points are the mean lengths for each repeat (*N* = 12, 7, and 7). The error bar is the SD of the mean lengths.

We next wondered whether CLASP2α could facilitate dynamic actin polymerization along the microtubule lattice. To probe this, we introduced 250 nM soluble, Alexa 647–labeled, globular actin (G-actin) into a flow cell containing Taxol-stabilized, coverslip-bound microtubules (Figure 3). In the presence of CLASP2α, we observed the binding and growth of dynamic actin filaments along the microtubule lattice, which continued to grow off the ends of the microtubule polymer. (Figure 3, A and B). Although individual microtubules were sparsely distributed on the coverslip surface, we often observed growing F-actin forming connections between microtubule polymers (Figure 3A; Supplemental Video 2). We measured the length of the individual F-actin connections between microtubules to be 7 ± 3 µm (SD, *N* = 26 F-actin bridges over three independent experimental days; Figure 3C). Thus, under these conditions, CLASP2α can promote linking of microtubules by actin filaments. Without CLASP2α, microtubules were not covered by F-actin and no connections were observed (Figure 3A). These results demonstrate that CLASP2α can support dynamic F-actin organization templated by microtubules and that F-actin can bridge microtubules, forming an interconnected network.

**Figure:** Movie S2 Dynamic actin filaments form bridges between microtubules over time. 30-minute timelapse merged images of Taxol microtubules and actin. Video playback is 25 frames per second.

### CLASP depletion results in disorganized actin fibers in cultured vascular smooth muscle cells

**FIGURE 4:**
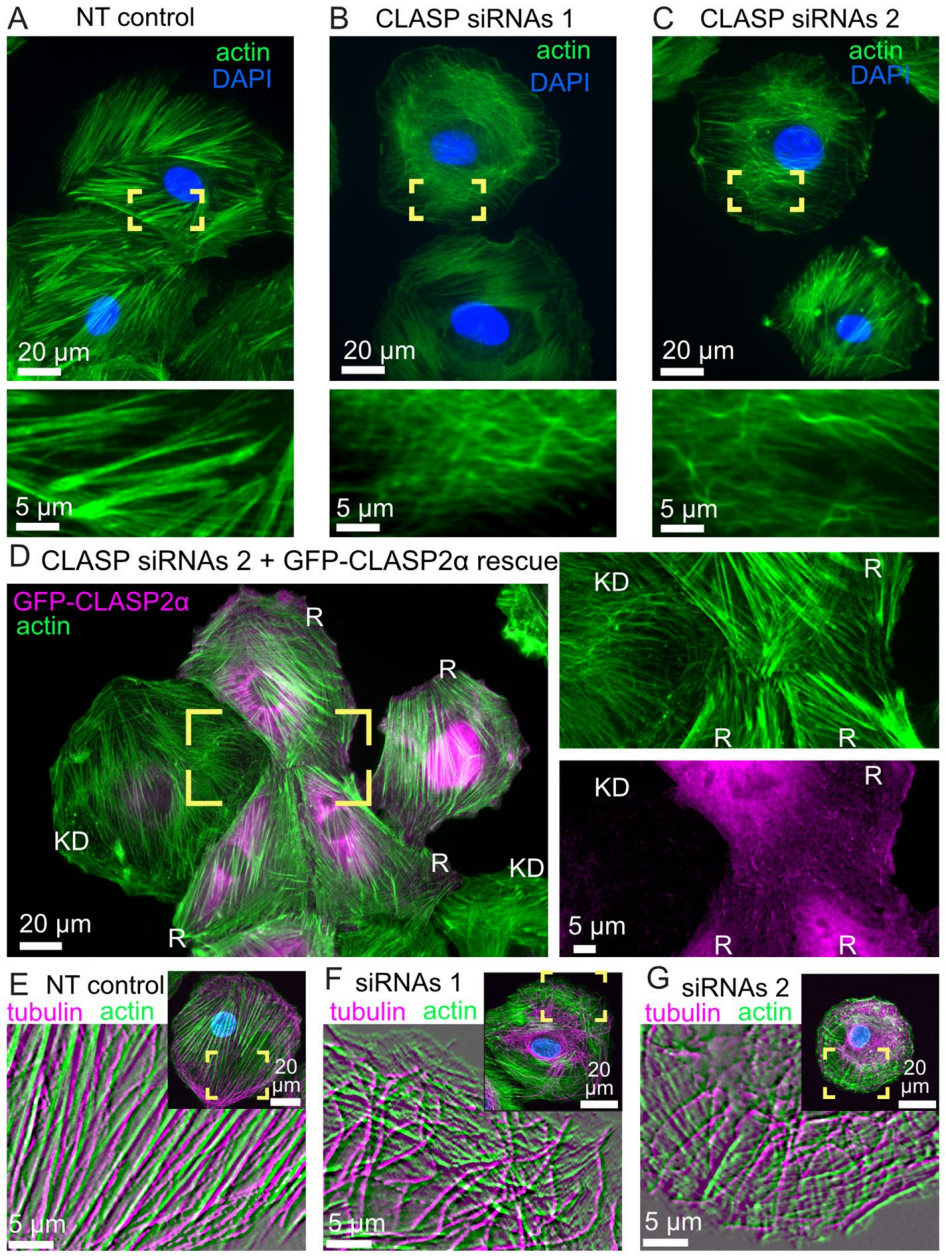
CLASPs are essential for correct stress fiber organization in vascular smooth muscle cells. (A) Prominent actin stress fibers in cells treated with a control (nontargeting, NT) siRNA. (B, C) Disorganized actin mesh in cells treated with two alternative combinations of siRNA oligos. Phalloidin-stained actin, green. DAPI, blue. Yellow boxes are enlarged to highlight details. The phenotypes have been vetted by a double-blinded evaluation (see *Materials and Methods*). (D) Cells treated with siRNA combination 2 and transfected with GFP-CLASP2 (pseudo-colored magenta) resistant to this siRNA. Separate channels from the yellow box are enlarged at the right. Actin organization in GFP-CLASP2–rescued cells (R) is like control (as in panel A), while in a nonexpressing cell (KD) it is like knockdown phenotype (as in panel C). Phalloidin-stained actin, green. GFP-CLASP2, magenta. (E, F) Actin and microtubules in cells treated with a control (nontargeting, NT) siRNA (E) or with siRNA combination 1 (F) or 2 (G). Phalloidin-stained actin, green. Tubulin, magenta. DAPI, blue. Yellow boxes in overview images are enlarged in insets. Inset images are processed through the emboss filter for exclusive visualization of fibers and illustration of their alignment with microtubules, which is diminished in F and G as compared with E. Scale bars, 20 μm in overviews and 5 μm in insets. All panels show representative images out of more than 45 cells per condition over three or more repeated experiments.

To probe CLASPs’ role in actin organization in cells, we depleted CLASPs in a rat vascular smooth muscle cell line, A7r5, chosen due to its notable actin bundle (stress fiber) structures (Figure 4A). For prominent phenotypes, both CLASP paralogues (CLASP1 and CLASP2) were depleted by small interfering RNA (siRNA) oligo combinations, carefully validated in previous work (Efimova *et al.*, 2014; Zhu *et al.*, 2016), achieving reliable reduction of CLASP protein levels (Supplemental Figure S2). Our results showed that actin fiber organization was severely disturbed in CLASP-depleted cells (Figure 4, B and C). Actin organization was efficiently rescued by ectopic overexpression of a nonsilenceable mutant of CLASP2 in depleted cells (Figure 4D), indicating that the CLASP2 paralogue is likely sufficient for proper actin organization in this context. Interestingly, coalignment of actin fibers with microtubules in CLASP-depleted cells (Figure 4, F and G) was reduced as compared with controls (Figure 4E), suggesting a diminished coordination of these filaments, consistent with our in vitro results. Overall, our findings suggest that CLASPs contribute to coorganization of higher-order actin structures with microtubules in cells.

## CONCLUSIONS

**FIGURE 5:**
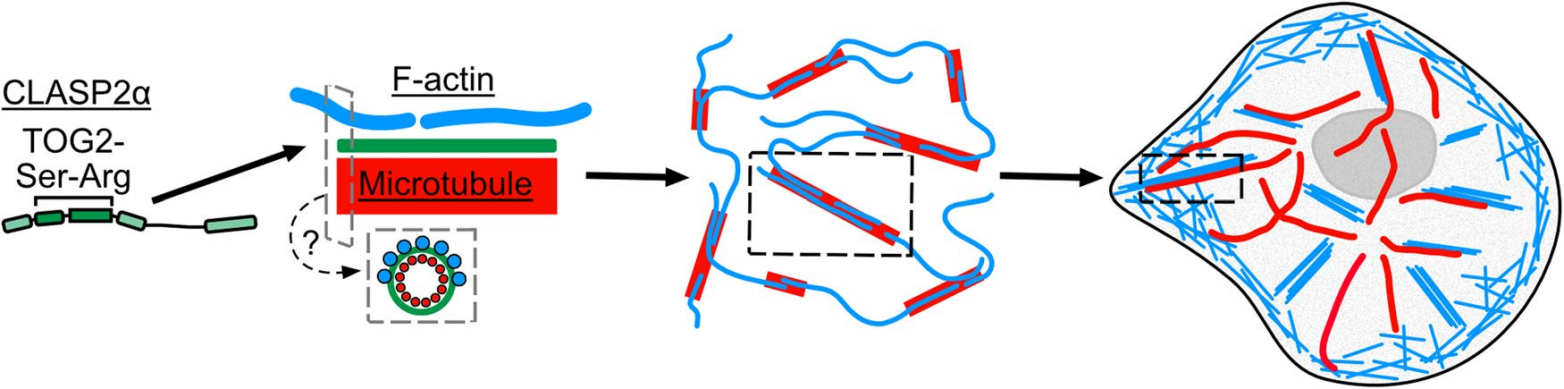
CLASP2 facilitates dynamic actin filament organization along the microtubule lattice. Microtubules are in red, actin is in blue, and CLASP2 is green throughout figure. Gray dashed boxes represent cross-sectional view of a potential model for F-actin organization along microtubules coated with CLASP2α, where F-actins are binding around the microtubule shaft. Black dashed boxes are highlighted zoom-ins representing instances of coorganization of actin filaments and microtubules observed in vitro and in cells.

In summary, our results demonstrate that CLASP2α directly cross-links F-actin to microtubules. We find that a minimal CLASP construct, containing the TOG2 domain and the serine-arginine–rich region of CLASP2, is sufficient for this cross-linking activity (Figure 5). The F-actin–microtubule cross-linking is achieved in spite of the relatively weak direct interaction between L-TOG2-S and F-actin. We propose that the strong interaction with microtubules concentrates L-TOG2-S (as well as full-length CLASP2α) along the microtubule lattice, thus providing a local abundance of weak F-actin binding sites and ultimately facilitating robust microtubule–CLASP–F-actin cross-linking.

Interestingly, the serine-arginine–rich region contained within the minimal L-TOG2-S was previously implicated in microtubule binding (Aher *et al.*, 2018). Furthermore, the same serine-arginine region contains a Ser-x-Ile-Pro (SxIP) motif, which encodes CLASP’s direct interaction with EB proteins, facilitating targeting of CLASP to growing microtubule ends (Patel *et al.*, 2012; Maki *et al.*, 2015). How interactions of the serine-arginine region with microtubules, actin, and EBs are regulated in different cellular contexts is not known. A previous report using coimmunoprecipitation and colocalization experiments in fibroblasts suggested that the TOG1 domain of CLASP2α also interacts with F-actin (Tsvetkov *et al.*, 2007). Our results show that the TOG1 domain is not necessary for the CLASP-mediated cross-linking of microtubules with F-actin; to what extent the TOG1 domain may contribute to direct CLASP–actin interaction warrants further investigation. Notably, a recent report suggested that another TOG-domain protein, XMAP215, directly interacts with F-actin to promote microtubule–actin coalignment in the neuronal growth cone (Slater *et al.*, 2019). There, the authors found that all five TOG domains of XMAP215 were required for XMAP215’s localization to F-actin. Future work is needed to determine the affinities of individual TOG domains for F-actin and their effects on the microtubule–actin cross-talk.

Our finding that microtubule-associated CLASP2α supports the binding of up to 11 actin filaments to a single microtubule region raises the question of F-actin organization on the microtubule lattice. Our results do not show any evidence of actin bundling along microtubules. The number of F-actin landing events saturated within the duration of our experiment, and we never observed more than 11 sequential landing events, suggesting a limit to the number of F-actin that can be linked to the microtubule lattice. Furthermore, we found no evidence of CLASP2α being able to directly bundle F-actin. This is different from the effects of another well-studied MAP, tau, which directly bundles F-actin and facilitates actin elongation and bundling along growing microtubules (Elie *et al.*, 2015). Given that Taxol-stabilized bovine microtubules typically contain 13 individual protofilaments (Amos and Klug, 1974; Arnal and Wade, 1995) and that the full microtubule surface may not be accessible to F-actin binding in our experiments (due to the tethering of microtubules to the coverslip), the number of landing events we observed is consistent with the model in which individual actin filaments bind around the microtubule perimeter along microtubule protofilaments (Figure 5). High-resolution structural approaches, such as cryo-electron microscopy, are needed to determine the exact organization of F-actin on CLASP2α-coated microtubules.

Our results demonstrate that CLASP2α-coated microtubules can template dynamic F-actin organization, specifically by F-actin linking multiple microtubules and forming an interconnected cytoskeletal network. Other cross-linking proteins have been shown to organize F-actin in a microtubule-centric manner, with notable examples belonging to the spectraplakin protein family (Rodriguez *et al.*, 2003; Suozzi *et al.*, 2012; Dogterom and Koenderink, 2019; Pimm and Henty-Ridilla, 2021). Interestingly, a number of microtubule plus-tip interacting proteins (+TIP) have also been shown to interact with F-actin and influence actin dynamics. Cytoplasmic linker interacting protein 170 (CLIP-170), a prominent +TIP and a known binding partner of CLASP, was recently reported to directly bind to F-actin and microtubules; however, the interaction of CLIP-170 with F-actin and microtubules was found to be mutually exclusive (Wu *et al.*, 2021). Similarly, EB1 was reported to directly interact with F-actin; however, it did not bind F-actin and microtubules simultaneously (Alberico *et al.*, 2016). While some of the +TIPs might not be able to directly cross-link F-actin and microtubules, many interactions among +TIPs in combination with other actin binding proteins result in the collective regulation of microtubule–actin cross-talk. For example, the combination of CLIP-170 and EB1 with actin regulators formin, mDia1, and profilin mediates F-actin polymerization from growing microtubule ends in vitro (Henty-Ridilla *et al.*, 2016). In another example, the +TIP protein adenomatous polyposis coli (APC) was reported to promote actin assembly, activity that is negatively regulated by interaction of APC with EB1 (Juanes *et al.*, 2020). Future work investigating how CLASP’s interactions with its binding partners may impact the cross-talk between the microtubule and actin networks is needed. It remains unclear whether CLASP can interact with F-actin when targeted to growing microtubule ends via EBs and whether such interaction could also promote actin polymerization from the dynamic microtubule ends, as shown for the complex with CLIP-170 (Henty-Ridilla *et al.*, 2016).

Microtubule-dependent actin filament coalescence demonstrated in our minimal component system could be important for building higher-order actin structures in cells. Regulators such as APC have been shown to both mediate actin assembly and facilitate microtubule capture at focal adhesions (Wen *et al.*, 2004; Juanes *et al.*, 2017, 2019). More recently, it has also been reported that APC can organize branched actin networks on microtubules in growth cones of hippocampal neurons (Efimova *et al.*, 2020). In our study, we find that both actin–microtubule association and the architecture of contractile actin fibers are severely disrupted when CLASPs are depleted in vascular smooth muscle cells. Actin defects observed in actin stress fiber structures are consistent with previous findings that CLASP depletions cause defects in actin-based invasive protrusions in vascular smooth muscle cells (Efimova *et al.*, 2014; Zhu *et al.*, 2016). Overall, our results identify a potential role for CLASP-mediated organization of actin filaments along microtubules in the process of actin fiber assembly in contractile cells.

## MATERIALS AND METHODS

### DNA constructs

The cDNA encoding full-length human CLASP2a was purchased from Dharmacon (accession: BC140778.1) and subcloned into a modified pHAT vector containing an N-terminal 6xHis tag and a C-terminal eGFP and StrepII tag (a gift from S. Bechstedt and G. Brouhard, McGill University, Canada). The cDNA encoding His-EGFP-L-TOG2-S in a pRSETa vector was a gift from E. Grishchuk (University of Pennsylvania, Philadelphia, PA).

### Protein biochemistry

#### His-CLASP2α-EGFP-Strep.

His-CLASP2α-EGFP-Strep was expressed in baculovirus-infected Sf9 insect cells using the Bac-to-Bac system (Invitrogen). After the first amplification, baculovirus-infected insect cell (BIIC) stocks were used to infect Sf9 cells at a density of 1 × 10^6^ viable cells/ml at a ratio of 10^–4^ BIIC:total culture volume (Wasilko and Lee, 2006; Wasilko *et al.*, 2009). Cells were harvested 5 d after infection. The cell pellets were lysed by one freeze–thaw cycle and Dounce homogenizing in lysis buffer containing 50 mM Tris (pH 7.5), 100 mM NaCl, 5% glycerol (vol/vol), 0.1% (vol/vol) Tween-20, 1 mM dithiothreitol (DTT) and supplemented with protease inhibitors. Genomic DNA was sheared by passing the lysate through an 18-gauge needle. The crude lysates were clarified by centrifugation for 20 min at 4°C and 35,000 rpm in a Beckman L90K Optima and 50.2 Ti rotor. Clarified lysates were applied to a HisTrapHP column (GE Lifesciences) according to the manufacturer’s protocol. His-CLASP2α-EGFP-Strep was eluted from the HisTrap column with 50 mM HEPES, pH 7.5, 150 mM NaCl, 5% glycerol, 0.1% Tween-20, 2 mM MgCl_2_, 1 mM DTT, 100 mM l-Glut/l-Arg, and a linear gradient of 50–500 mM imidazole. His-CLASP2α-EGFP-Strep protein was then further purified by size exclusion chromatography using a Superdex 200 Increase 10/300 GL column (Cytiva) in CLASP storage buffer containing 25 mM PIPES (piperazine-*N*,*N*′-bis(2-ethanesulfonic acid)) (pH 6.8), 150 mM KCl, 5% (vol/vol) glycerol, 0.1% (vol/vol) Tween-20, 50 mM l-glutamate, 50 mM l-arginine, and 1 mM DTT.

#### His-EGFP-L-TOG2-S.

His-EGFP-L-TOG2-S was expressed in BL21(DE3) *Escherichia coli* cells. Expression was induced with 0.2 mM Isopropyl β-d-1-thiogalactopyranoside (IPTG) at 18°C for 16 h. Cells were lysed for 1 h at 4°C in 50 mM HEPES (pH 7.5), 300 mM NaCl, 2 mM MgCl_2_, 5% (vol/vol) glycerol, 0.1% (vol/vol) Tween-20, 1 mM DTT, and 40 mM imidazole supplemented with 1 mg/ml lysozyme, 10 mg/ml phenylmethylsulfonyl fluoride (PMSF) and EDTA-free protease inhibitors (Roche). The crude lysate was sonicated on ice and then clarified by centrifugation for 30 min at 4°C and 35,000 rpm in a Beckman L90K Optima and 50.2 Ti rotor. Clarified lysates were applied to a HisTrapHP column (Cytiva) according to the manufacturer’s protocol. His- EGFP-L-TOG2-S protein was eluted with 50 mM HEPES (pH 7.5), 500 mM NaCl, 2 mM MgCl_2_, 5% (vol/vol) glycerol, 0.1% (vol/vol) Tween-20, and 1 mM DTT and linear gradient of 40–500 mM imidazole. Purified His-EGFP-L-TOG2-S from the HisTrap column was desalted into CLASP storage buffer using a PD-10 desalting column (Cytiva).

All proteins were snap frozen in single-use aliquots, and protein purity was assessed by 10% SDS–PAGE (Bio-Rad Laboratories) and stained with Coomassie Brilliant Blue (Supplemental Figure S1).

#### Tubulin purification and labeling.

Tubulin was purified from bovine brains using the standard protocol (Castoldi and Popov, 2003). Briefly, tubulin was purified by cycles of polymerization and depolymerization using the high-molarity PIPES buffer method (Castoldi and Popov, 2003). Tubulin was labeled with Alexa Fluor 555 and 647 dyes (Invitrogen) and biotin (Sigma-Aldrich) following a published protocol (Hyman *et al.*, 1991).

#### Actin purification and labeling.

Actin was purified from chicken breast using the standard protocol (Pardee and Spudich, 1982). First, the chicken breast was made into a muscle acetone powder and then further purified through cycles of polymerization and depolymerization and stored in general actin buffer (2 mM Tris-HCl, pH 8.0, 0.2 mM ATP, 0.5 mM DTT, 0.1 mM CaCl_2_, and 1 mM NaN_3_) (Pardee and Spudich, 1982). Actin was labeled with Alexa Fluor 647 (Invitrogen) following a published protocol (Shekhar, 2017). Actin was flash-frozen and stored at –80°C. Before actin dynamics experiments, actin was left on ice overnight and preclarified by spinning at 450,000 × *g* for 20 min at 4°C in a TL Optima ultracentrifuge and stored at 4°C for up to 3 wk.

### Cosedimentation experiments

F-actin was prepared by first preclarifying G-actin, stored in general actin buffer, by spinning the sample at 13,300 rpm at 4°C for 15 min. Supernatant was collected and added to an actin polymerization buffer (50 mM KCl, 2 mM MgCl_2_, and 1 mM ATP) and was incubated for 1 h at room temperature to obtain F-actin. GFP-L-TOG2-S protein was preclarified by spinning down at 400,000 × *g* for 10 min at 4°C. A titration of F-actin concentrations (0, 0.5, 1, 2.5, 5, and 10 µM) were incubated with 360 nM GFP-L-TOG2-S at 37°C for 15–30 min in general actin buffer. Then, samples were ultracentrifuged (Optima-MAX; Beckman Coulter) at 184,000 × *g* for 20 min at 26°C. Top supernatant (50 µl) was collected, and 13 µl of 5× SDS loading dye was added. The remaining supernatant was collected and stored as waste. The pellet was resuspended in 63 µl of 1× SDS loading dye and subsequently stored at –20°C. Samples were then prepared for electrophoresis. The pellet sample was thawed, and 15 µl of sample was added to 15 µl of 1× SDS loading dye. Top supernatant sample (30 µl) was thawed, and then samples were boiled at 95°C for 5 min and then loaded onto a 12% SDS–PAGE gel. Gels were run at 50 V for 15 min and then 150 V for at least 1 h. Subsequently, gels were fixed with fix solution (50% methanol, 7% acetic acid), rocking twice for 30 min at room temperature. Then, gels were stained with SYPRO Ruby Protein Gel Stain (Invitrogen) overnight. Gels were then washed with wash solution (10% methanol, 7% acetic acid) for 30 min, rocking at room temperature. Finally, gels were washed twice for 5 min with Milli-Q H_2_O, rocking at room temperature, and imaged with a Gel Doc EZ system (Bio-Rad). Experiments were performed in triplicate.

### In vitro reconstitution assay conditions and imaging

#### Chamber preparation.

Microscope chambers were constructed as previously described (Gell *et al.*, 2010; Strothman *et al.*, 2019). Channels were constructed by sandwiching 22 × 22 mm and 18 × 18 mm silanized glass coverslips together between three thin strips of parafilm. A heat block was used to melt the parafilm and stick the coverslips together. The surface was functionalized by flowing in 25–100 µg/ml NeutrAvidin (Thermo Scientific) for 10 min, followed by blocking with 1% Pluronic F127 for 30 min. Chambers were washed in between these steps using MRB80 or BRB80 (80 mM PIPES, 1 mM EGTA, and 4 or 1 mM MgCl_2_, respectively, pH 6.8).

#### Imaging.

Imaging was performed using TIRF on a Nikon Eclipse Ti microscope with a 100×/1.49 n.a. TIRF objective, equipped with an Andor iXon Ultra EM-CCD camera, 488-, 561-, and 640-nm solid state lasers (Nikon Lu-NA), Finger Lakes Instruments HS-625 high-speed emission filter wheel, and standard filter sets. Laser exposure time was 100 ms for all experiments. Experiments were performed at 35°C using a Tokai Hit objective heater. Images were acquired using NIS-Elements (Nikon).

#### F-actin binding along CLASP2-coated microtubule experiments.

Taxol microtubules were prepared by polymerizing 28 µM tubulin (16% biotinylated and 5% Alexa 647–labeled) with a microtubule polymerization mix (MRB80, 5% dimethyl sulfoxide (DMSO), 4 mM MgCl_2_, and 1 mM GTP). The reaction was left on ice for 5 min and then incubated for 1 h at 37°C. Then, microtubules were diluted 56 times into 10 µM Taxol (Tocris) in MRB80 (MRB80T) while in the heat block. Microtubules were then spun down in a Beckman Airfuge IM-13 at 20 psi for 5 min at room temperature. Pellet was resuspended in 100 µl of MRB80T. Microtubules were stored in the dark at room temperature and used within 1 wk. Phalloidin-stabilized F-actin was prepared by polymerizing 3.7 µM unlabeled G-actin, stored in the general actin buffer, with 50 mM KCl, 1 mM MgCl_2_, and 1 mM ATP for 1 h at room temperature. Then, equimolar TRITC-phalloidin (Sigma-Aldrich) was added to the reaction mix and incubated in the dark for 15 min at room temperature. F-actin was then spun down in an airfuge at 27 psi for 10 min. Pellet was resuspended in MRB80, 0.2 mM ATP, and 0.5 mM DTT. F-actin was stored at 4°C and used for 1 wk. For control experiments with and without phalloidin stabilization, F-actin was prepared similarly as above, but was polymerized with 20% labeled A647 G-actin for 1 h at room temperature and then the reaction was split into two, where one was stabilized with equimolar unlabeled phalloidin (Sigma-Aldrich) and the other half used in parallel.

Taxol microtubules were added to NeutrAvidin-functional channels to bind to the surface. Once bound after a few minutes, channel was washed with MRB80T and then washed with imaging buffer (MRB80T, 0.2 mM ATP, 40 µg/ml glucose oxidase, 40 mM glucose, 16 µg/ml catalase, 0.08 mg/ml casein, and 10 mM DTT). Then, reaction mixes with imaging buffer and either 100 nM CLASP2α-GFP or 100 nM L-TOG2-S and 6.5 µM TRITC-phalloidin–stabilized F-actin were added to the imaging channel while simultaneously imaging. Control experiments were performed with CLASP storage buffer. Control experiments with and without phalloidin stabilization were performed with 1 µM F-actin. All solutions were supplemented with 20 µM Taxol.

For the actin filament and microtubule colocalization experiments, CLASP2α-GFP or GFP-L-TOG2-S reaction mixes with F-actin were added to the channel and then images of all three channels (488 nm for CLASP protein constructs, 561 nm for TRITC-phalloidin F-actin, and 640 nm for 5% A647-labeled microtubules) were acquired every 3 s for 10 min. Several images were taken throughout the channel after the 10-min incubation.

For F-actin accumulation experiments, a three-color image to visualize the CLASP2α-GFP (488 nm), TRITC-phalloidin F-actin (561 nm), and microtubules (640 nm) was taken before adding the F-actin-CLASP reaction mix as a control for fluorescence bleed through. Then, fast imaging at 5 fps for the 561-nm channel was started while flowing in the reaction mix to capture the initial F-actin landing events. After 5 min, another three-color image to visualize the CLASP (488 nm), F-actin (561 nm), and microtubule (640 nm) channels was taken and then 488- and 561-nm channels were imaged every 3 s for 35 min. Once done, a final three-color image was taken. Experiments were performed in triplicate. Control experiments with and without phalloidin were done in duplicate.

#### F-actin/microtubule bundling experiments.

TRITC-phalloidin F-actin (2.8 µM) and Taxol-stabilized microtubules (2.5 µM, 24% TAMRA labeled) were prepared as described above; however, coverslips were passivated with a TRITC antibody. Reaction mixes with imaging buffer (MRB80, 0.2 mM ATP, 40 µg/ml glucose oxidase, 40 mM glucose,16 µg/ml catalase, 0.08 mg/ml casein, and 10 mM DTT), 1 µM F-actin, and 200 nM fascin (Cytoskeleton) or 200 nM CLASP2α-GFP or CLASP2α-GFP storage buffer were incubated at room temperature for 10 min. As a positive control, 200 nM CLASP2α-GFP or storage buffer was incubated with 1 µM Taxol microtubules, imaging buffer, and 10 µM Taxol at room temperature for 10 min. Then, reaction mixes were added to an imaging channel and then immediately washed with imaging buffer. Ten to twelve TIRF images were taken every 750 µm along the channel. The 488-nm channel was used to visualize CLASP2α-GFP, and the 561-nm channel was used to visualize F-actin or microtubules. Experiments were done in triplicate.

#### Actin dynamics on CLASP2-coated microtubule experiments.

Taxol microtubules were prepared as described above; however, they were labeled with 16% biotin and 5% Alexa 555 tubulin and stored in BRB80T (BRB80, 10 µM Taxol [Tocris]). After chamber preparation, Taxol microtubules were added to bind to the surface and then washed out with BRB80T. Before adding the reaction mix, the chamber was washed with imaging buffer (BRB80, 0.1% methylcellulose, 40 µg/ml glucose oxidase, 40 mM ᴅ-glucose, 18 µg/ml catalase, 0.8 mg/ml casein, 10 mM DTT, and 0.2 mM ATP). Then, either 100 nM CLASP2α-GFP or CLASP2α-GFP storage buffer in imaging buffer was added to the chamber and incubated for 5 min to allow for full coating of Taxol microtubules. Then a reaction mix with 250 nM, 20% Alexa 647–labeled G-actin and either 100 nM CLASP2α-GFP or CLASP2α-GFP storage buffer were added. Three-color imaging every 5 s for 30 min immediately followed. All solutions were supplemented with 20 µM Taxol. Experiments were done in triplicate.

### Cells

A7r5 rat smooth muscle cells (American Type Culture Collection #CRL-1444) were grown in low-glucose (1000 mg/l) DMEM without Phenol Red, supplemented with 10% fetal bovine serum at 37°C and 5% CO_2_. Cells were plated on glass coverslips coated with 10 µg/ml fibronectin 24 h before experiments.

#### siRNA sequences, CLASP2 expression rescue, and transfection.

Two different combinations of mixed siRNA oligonucleotides against CLASP1 and CLASP2 were used. Combination 1 (custom design; Sigma) included the CLASP1-targeted siRNA sequence 5′-CGGGAUUGCAUCUUUGAAA-3′ and the CLASP2-targeted siRNA sequence 5′- CUGAUAGUGUCUGUUGGUU-3′. Combination 2 (Mimori-Kiyosue *et al.*, 2005) included the CLASP1-targeted siRNA sequence 5′-CCUACUAAAUGUUCUGACC-3′ and the CLASP2-targeted siRNA sequence 5′-CUGUAUGUACCCAGAAUCU-3′. Nontargeting siRNA (Dharmacon) was used for controls. For siRNA oligonucleotide transfection, HiPerFect (Qiagen) was used according to the manufacturer’s protocol. Experiments were conducted 72 h after siRNA transfection, as at this time minimal protein levels were detected.

For CLASP2 expression rescues, a GFP-labeled CLASP2 mutant using alternative codons and therefore nonsilenceable by anti-CLASP2 siRNA from combination 2 (Mimori-Kiyosue *et al.*, 2005) was transfected into depleted cells at 48 h after siRNA transfection. For DNA transfection, Fugene6 (Roche) was used according to the manufacturer’s protocol. Cells were processed for imaging after an additional 24 h to meet the 48-h depletion optimum.

#### Cell labeling, imaging, and image processing.

For actin imaging, cells on coverslips were fixed in 4% paraformaldehyde plus 0.3% Triton X-100 in cytoskeleton buffer (10 mM MES, 150 mM NaCl, 5 mM EGTA, 5 mM glucose, and 5 mM MgCl_2_, pH 6.1) for 10 min. For costaining with tubulin, 0.1% glutaraldehyde was added into the fixative solution. The actin cytoskeleton was visualized by phalloidin conjugated to Alexa Fluor 488 (Invitrogen, Molecular Probes). Tubulin was immunostained using anti–α-tubulin monoclonal antibodies DM1a (Sigma-Aldrich) and Alexa 568–conjugated goat anti-mouse immunoglobulin G antibodies (Invitrogen, Molecular Probes) as secondary antibodies. CLASPs were immunostained by non–paralogue-specific rabbit polyclonal antibodies VU-83 (pan-CLASP antibodies) (Efimov *et al.*, 2007). Nuclei were visualized by diamidino-2-phenylindole (DAPI; ThermoFisher). Staining was performed at room temperature. After being washed, samples were mounted into ProLong Gold Antifade Reagent (Invitrogen, Molecular Probes) on glass slides and stored at −20°C.

Wide-field fluorescence imaging (Figure 4, A–D; Supplemental Figure 2, A–C) was performed using a Nikon 80I microscope with a CFI APO 60× oil lens, NA 1.4, and CoolSnap ES CCD camera (Photometrics). Laser-scanning confocal imaging was performed using Nikon A1r based on a Ti-E inverted microscope with SR Apo TIRF 100× NA 1.49 oil lens run by NIS Elements C software (Nikon, Tokyo, Japan). (Figure 4, E–G) Maximum-intensity projection over the whole cell is shown in overview images. Single confocal slices processed through the emboss filter are shown in insets. In all cell images, each fluorescence channel was contrasted by whole-image histogram stretching. In overview images in Figure 4, E–G, the tubulin channel was gamma-adjusted to highlight microtubules at the cell periphery.

#### Phenotype verification.

Actin channel images (as in Figure 4, A–C) were separated and coded for double-blind phenotype verification. Three researchers independently sorted images to detect actin fiber disturbance. These blinded analyses resulted in >84% of correct image designation into NT control and CLASP-depleted phenotypes.

#### CLASP Western blotting.

Western blotting was performed using the Protein Electrophoresis and Blotting System (Bio-Rad). Briefly, A7r5 cells were transfected with two different combinations of mixed siRNA oligonucleotides against CLASP1 and CLASP2 using TransIT-X2 (Mirus). After 72 h, the cells were collected, lysed, and resuspended in Laemmli Sample Buffer (Bio-Rad). Total protein for each condition (20 µg) was resolved on 12% SDS–PAGE gels and transferred to nitrocellulose membranes (GE Healthcare Life Sciences) at 350 mA for 3 h for blotting. The membranes were then blocked with 5% nonfat dried milk (Sigma-Aldrich) for 1 h and incubated overnight with primary antibodies: anti-rat CLASP1 (KT 67, Absea), anti-rat CLASP2 (KT69, Absea), and anti-mouse GAPDH (Santa Cruz). IRDye 700 or 800 (LI-COR Biosciences) was used as secondary antibody. The membranes were imaged on an Odyssey Infrared Imaging system (LI-COR Biosciences).

### Image analysis

Images were processed using the Fiji (Fiji Is Just another ImageJ) image processing package (Schindelin *et al.*, 2012). Figures were prepared in Affinity Designer (Serif).

#### Protein gel densitometry.

Gels were quantified using the Gel Analyzer ImageJ plug-in. In brief, lane profile plots were produced using the rectangular selection tool, lines were drawn to enclose the peaks of interest, and the peak areas were measured in triplicate. The top band of the GFP-L-TOG2-S protein was measured. The peak area measurements for the GFP-L-TOG2-S protein were averaged. Then, each supernatant and pellet peak areas were summed, and the peak area of the GFP-L-TOG2-S in the pellet sample was divided by the total amount of protein in the sample. Then, the fraction of GFP-L-TOG2-S in the pellet was plotted as a function of F-actin concentration and fitted to the Michaelis–Menten equation,




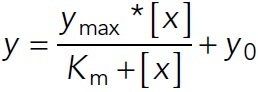

1



using the MATLAB Curve Fitting Tool (MathWorks). The *y*_max_*, y*_0_*,* and *K_m_* values were calculated with the 95% confidence interval. The resulting fit was plotted with the individual data points in MATLAB. Error bars on the individual data points are the error propagation of standard deviations from averaged measurements.

#### Colocalization using PCC.

Three, three-color images of 488-nm (Control, CLASP2α-GFP, or GFP-L-TOG2-S), 561-nm (TRITC-phalloidin F-actin), and 640-nm (5% A647-labeled microtubules) channels were cropped to 335 × 170 pixel size (∼54 µm × 27 µm). Then, the PCC was measured using the ImageJ plug-in, JACoP (Just Another Co-localization Plugin) for microtubules versus CLASP and microtubules versus F-actin (Bolte and Cordelières, 2006; Dunn *et al.*, 2011). Individual PCC values with the SD were plotted in GraphPad Prism. The Kruskal–Wallis one-way analysis of variance with multiple comparisons test was used to test for significance in GraphPad Prism. Experiments were done in triplicate.

#### Average actin intensity on CLASP2-coated microtubules.

All microscopy movies were first drift-corrected using the Image Stabilizer plug-in for ImageJ. To avoid heterogeneous illumination occurring in the TIRF field, all the microscope images were cropped (∼300 × 512 pixel size) to eliminate out-of-focus or dimmer microtubules. For F-actin landing experiments, the microtubule channel image taken between the first 5-min movie and the second 35-min movie was used to determine the microtubule region. For actin polymerization experiments, the last microtubule image was used. This image was thresholded, using the Auto MaxEntropy method, and recorded. Then a selection was created around the thresholded microtubule region and overlaid on the F-actin channel. A region of interest was created, and the average F-actin intensity was measured using the Time Series Analyzer V3 ImageJ plug-in. F-actin intensity was normalized to the maximum intensity and fitted to the following intensity equation:




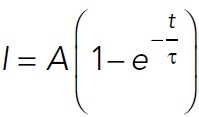

2



where *I* is the F-actin intensity, *A* is the maximum F-actin intensity, *t* is time, and τ is the half-time in the MATLAB Curve Fitting Tool (MathWorks). Results were plotted in MATLAB (MathWorks).

#### F-actin landing step analysis.

For kymograph production, straight or segmented lines were drawn along microtubules and then overlaid on the F-actin channel. Straight-line kymographs were produced using a custom ImageJ plug-in, and segmented-line kymographs (used occasionally for curvy microtubules) were produced using the KymographBuilder ImageJ plug-in. Kymographs were made for the first 5-min movies and the second 35-min movie along the same microtubule. Because F-actin landing events appear as steps, we used the open-source, vbFRET graphical user interface (GUI) to measure the number of “steps” (Bronson *et al.*, 2009). This software was created to analyze single-molecule fluorescence resonance energy transfer (FRET) data using hidden Markov modeling and finds the most probable fit using the variational Bayesian expectation maximization algorithm (Bronson *et al.*, 2009). For analysis, kymographs were loaded into a custom MATLAB function that extracts vertical line scans of a designated width (3 pixels) and intensity values were normalized to 90% of the maximum intensity. This normalization was recommended when analyzing non–single molecule FRET data using the vbFRET software (Bronson *et al.*, 2009). To use this software, the FRET efficiency, which is defined as




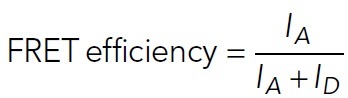

3



was set equal to the F-actin intensity or donor intensity (*I_D_*) by defining the acceptor intensity (*I_A_*) as




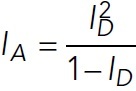

4



Line scans were selected based on the center-most line scan, and then additional line scans were selected in intervals of 15 pixels (2.5 microns) along the microtubule lattice, as permitted by individual microtubule lengths. Selected line scans were then loaded into the vbFRET GUI for stepping analysis (Bronson *et al.*, 2009). The number of possible states was set to a minimum of 1 and a maximum of 20, and the number of fitting attempts per trace was set to 25. The vbFRET session and idealized traces were saved, and steps were extracted using custom MATLAB codes. Steps were measured after F-actin was visible in solution (after 30–40 s). Owing to overfitting of noise, step fits were filtered, with steps of sizes smaller than the three SDs below the mean step size removed. F-actin landing events followed by F-actin unbinding (negative steps) were additionally removed to consider the maximum number of F-actin accumulating on a microtubule segment. Line traces were analyzed separately for 5- and 35-min movies and reported results are the summation of the two movies to ensure that all F-actin landing events are measured. The example F-actin intensity trace in Figure 2, with vbFRET-generated fit, was produced in MATLAB (MathWorks), and F-actin landing event numbers were plotted in GraphPad Prism.

#### F-actin/microtubule bundle quantification.

Images were cropped (273 × 285 square pixels or 43.7 × 45.6 square microns), and the 561-nm (F-actin or microtubule) channel was analyzed. First, the single-filament intensity was determined from the F-actin–alone and microtubule-alone images. This was determined by using the Adjust Threshold plug-in in ImageJ, where the “Auto” “Default” method was used to mask single filaments. The mean intensity in this area was measured and averaged with other images from the same day. From this measurement, we determined a minimum bundle intensity as defined by two times the intensity of the single filament. This value was determined for each experimental day. Using the “Default” threshold method, we thresholded intensities greater than the minimum bundle intensity for all images. Then, the Analyze Particles plug-in in ImageJ was used to identify F-actin and microtubule bundles. The minimum particle size was set to 60 square pixels, and the particle size, mean intensity, and SD were recorded for all conditions. An overlay mask was produced for each image to visualize particles determined as bundles. Results are represented as the number of fields of view that contained at least one F-actin or microtubule bundle particle.

#### F-actin bridge length.

After 30 min, F-actin bridge lengths were measured by drawing segmented lines along F-actin in ImageJ. Error bars are the SD of the mean F-actin bridge lengths for each repeat. Analysis and plots were done in Microsoft Excel and GraphPad Prism.

## Abbreviations used:

APC: adenomatous polyposis coli
CLASP: cytoplasmic linker–associated protein
F-actin: actin filaments
MAP: microtubule-associated protein
PCC: Pearson correlation coefficient
+TIP: microtubule plus tip–interacting protein
TIRF: total internal reflection fluorescence microscopy
TOG: tumor-overexpressing gene
TRITC: tetramethylrhodamine B isothiocyanate.
