# Supplementary material for: CLASP2 facilitates dynamic actin filament organization along the microtubule lattice
Source: Mol Biol Cell. 2023 Feb 21;34(3):br3. doi: 10.1091/mbc.E22-05-0149 (PMC10011731; doi:10.1091/mbc.E22-05-0149)
Supplement: Supplementary file 3 [file mbc-34-br3-s001.pdf]

Supplementary Materials

Molecular Biology of the Cell

Rodgers *et al*.

SUPPLEMENTAL MATERIALS

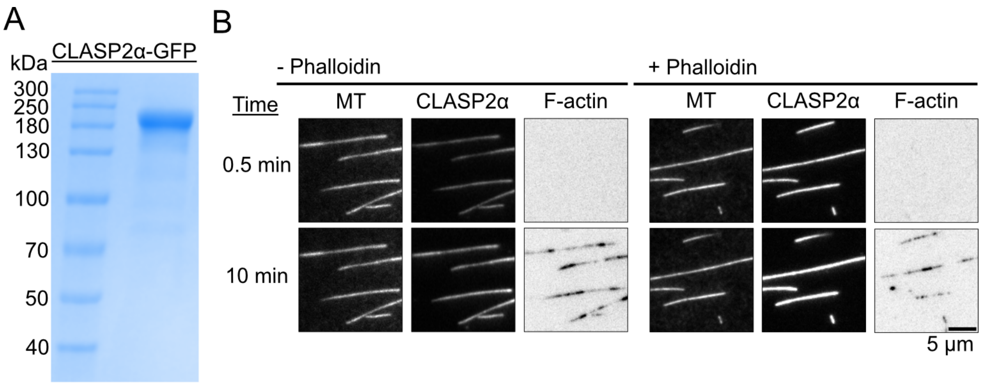

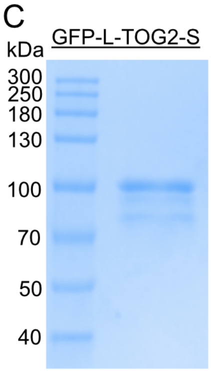

Accession	Description	% Coverage	Unique spectrum count	Total spectrum count	Mw (kDa)
N/A	EGFP-L_TOG2-S	70	119	346	91
P04264	Keratin II	52	38	38	66
P13645	Keratin I	39	24	25	59
P35527	Keratin I	37	21	24	62
P35908	Keratin II	33	19	21	65
P08779	Keratin I	28	5	14	51
P02533	Keratin I	22	8	13	52
P13647	Keratin II	16	8	11	62
P48668	Keratin II	15	3	10	60
P02538	Keratin II	16	1	10	60

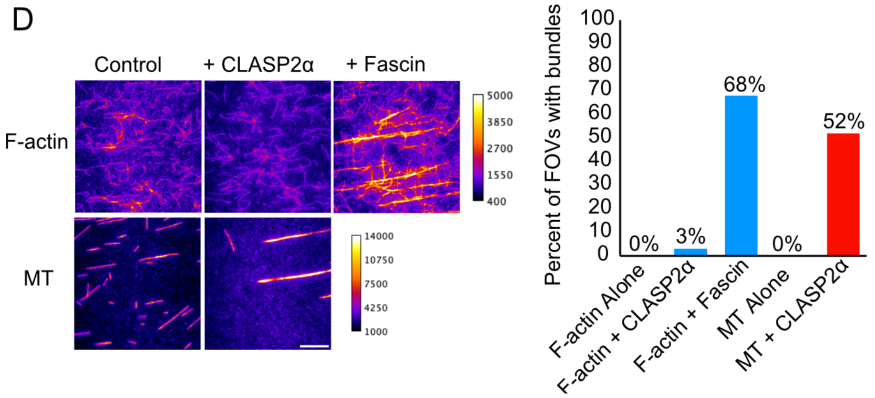

Supplemental Figure 1. Accumulation of multiple actin filaments on microtubules is not dependent on TRITC-phalloidin stabilization, and purified human CLASP2α does not bundle actin filaments. A) SDS-PAGE gel showing purified His-CLASP2α-eGFP-Strep. B) Example TIRF time-lapse images of 1 µM phalloidin-stabilized F-actin and 1 µM F-actin (20% Alexa647-labeled) landing along CLASP2α-coated microtubules. C) SDS-PAGE gel showing purified His-eGFP-L-TOG2-S. The lower band in the His-EGFP-L-TOG2-S sample likely represents a truncated protein or breakdown product as no significant contaminating proteins were found in mass spectroscopy analysis. Mass spectrometry analysis of His-EGFP-L-TOG2-S protein. The hits with a spectrum count of 10 or more are listed. D) Example TIRF images of F-actin or microtubule (MT) structures in control (alone), with 200 nM CLASP2α-GFP, or with 200 nM fascin. Scale is for heat map coloring. Quantification of the percent of field of views (FOVs) with at least one bundle as defined by intensity thresholding (see Methods). Experiments done in triplicate. F-actin alone, N = 29; microtubules alone, N = 24; F-actin + CLASP2α, N = 29; microtubules + CLASP2α, N = 27; and F-actin + fascin, N = 31 FOV.

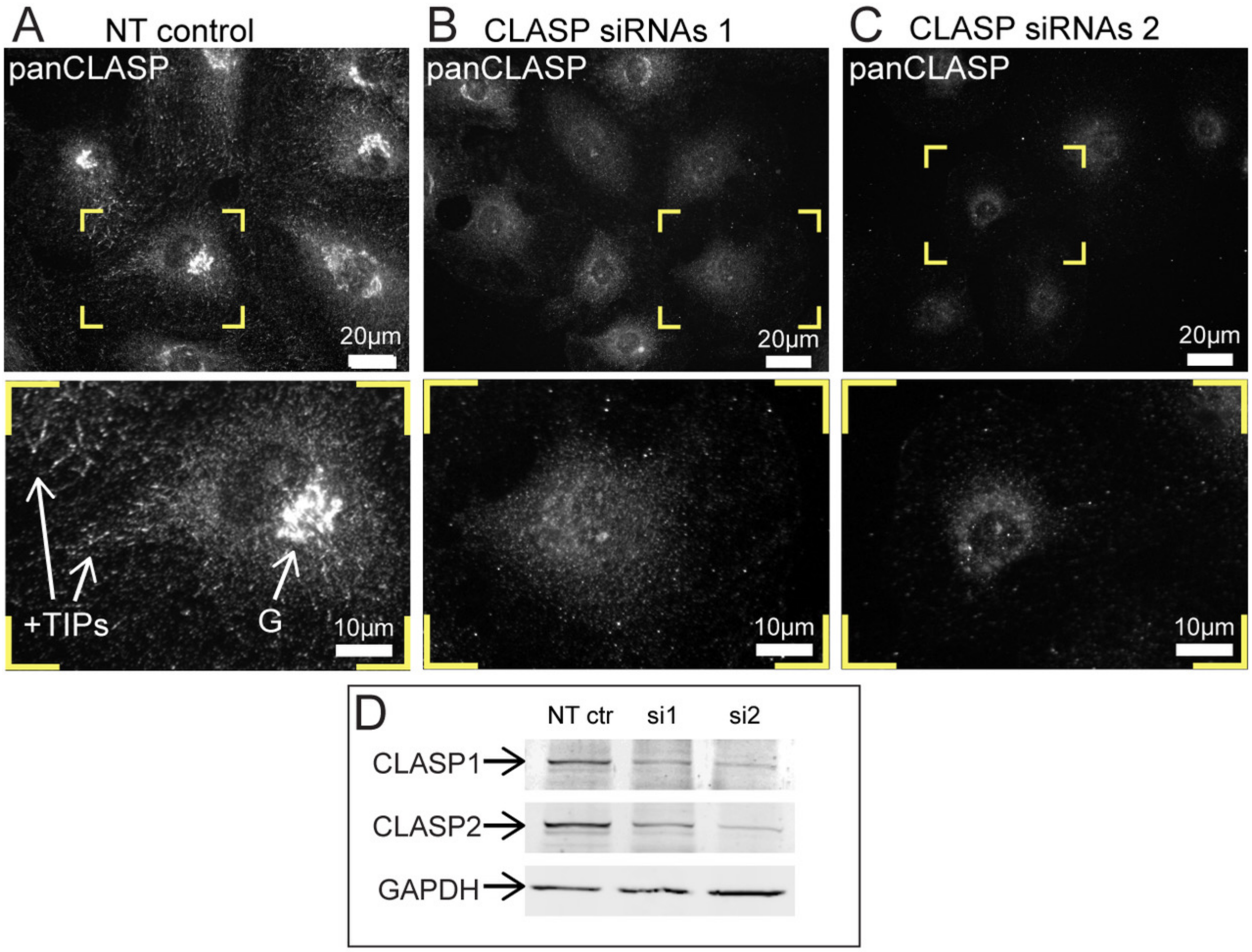

Supplemental Figure 2. CLASPs are efficiently knocked down by siRNA in A7r5 cells. A-C) immunostaining with pan-CLASP antibody (grayscale). A) NT control, B) siRNA combination 1, C) siRNA combination 2. Yellow boxes are enlarged below to highlight details. Scale bars, 20 µm in overviews and 10 µm in insets. D) Western blotting indicating CLASP1 and CLASP2 protein level reduction in cell population treated with siRNA combinations 1 and 2 as compared to control. Loading control, GAPDH.

Video 1. F-actin landing on CLASP2-coated microtubules. Time-lapse of 6.5 µM F-actin and three-color merged time-lapse. F-actin time-lapse is merged with still microtubule image and average projection of CLASP2α-GFP signal. Video playback is 300 frames per second.

Video 2. Dynamic actin filaments form bridges between microtubules over time. 30-minute time-lapse merged images of Taxol microtubules and actin. Video playback is 25 frames per second.
