# Supplementary material for: CLASP2 facilitates dynamic actin filament organization along the microtubule lattice
Source: Mol Biol Cell. 2023 Feb 21;34(3):br3. doi: 10.1091/mbc.E22-05-0149 (PMC10011731; doi:10.1091/mbc.E22-05-0149)
Supplement: Supplementary file 4 [file mbc-34-br3-s002.pdf]

CLASP2 facilitates dynamic actin filament organization along the microtubule lattice

Nicole Rodgers, Elizabeth Lawrence, Avishkar Sawant, Nadia Efimova, Gabriela Gonzalez-Vasquez, Tara Hickman, Irina Kaverina, and Marija Zanic

Corresponding author(s): Marija Zanic, Vanderbilt University

Review Timeline:

Submission Date:	2022-05-03
Editorial Decision:	2022-06-16
Revision Received:	2022-12-14
Accepted:	2022-12-17

Editor-in-Chief: Matthew Welch

Transaction Report:

(Note: With the exception of the correction of typographical or spelling errors that could be a source of ambiguity, letters and reports are not edited. The original formatting of letters and referee reports may not be reflected in this compilation.)

RE: Manuscript #E22-05-0149
TITLE: "CLASP2 facilitates dynamic actin filament organization along the microtubule lattice"

Dear Marija,

We have received feedback from two experts in the field. While they found your study interesting, they have some important points that you should address before publication in MBoC.

The specific points are:
From reviewer 1: Quantification of actin using fluorescent phalloidin; use of a high concentration of actin (6.5 µM) to study CLASP/actin interaction.
Regarding this later comment, could you determine the affinity of CLASP2 for the actin filament using the pelleting assay in SUP Figure 1 with a varying concentration of actin filaments?

From reviewer #2: A rescue experiment seems necessary to strengthen your siRNA experiments.
This reviewer also made an interesting comment about the actin-microtubule overlap in CLASP2 WT vs siRNA.

Sincerely,

Laurent Blanchoin
Monitoring Editor
Molecular Biology of the Cell

Dear Dr. Zanic,

The review of your manuscript, referenced above, is now complete. The Monitoring Editor has decided that your manuscript requires minor revisions before it can be published in Molecular Biology of the Cell, as described in the Monitoring Editor's decision letter above and the reviewer comments (if any) below.

A reminder: Please do not contact the Monitoring Editor directly regarding your manuscript. If you have any questions regarding the review process or the decision, please contact the MBoC Editorial Office (mboc@ascb.org).

When submitting your revision include a rebuttal letter that details, point-by-point, how the Monitoring Editor's and reviewers' comments have been addressed. (The file type for this letter must be "rebuttal letter"; do not include your response to the Monitoring Editor and reviewers in a "cover letter.") Please bear in mind that your rebuttal letter will be published with your paper if it is accepted, unless you have opted out of publishing the review history.

Authors are allowed 180 days to submit a revision. If this time period is inadequate, please contact us immediately at mboc@ascb.org.

In preparing your revised manuscript, please follow the instruction in the Information for Authors (www.molbiolcell.org/info-for-authors). In particular, to prepare for the possible acceptance of your revised manuscript, submit final, publication-quality figures with your revision as described.

To submit the rebuttal letter, revised version, and figures, please use this link (please enable cookies, or cut and paste URL):
Link Not Available

Authors of Articles and Brief Communications whose manuscripts have returned for minor revision ("revise only") are encouraged to create a short video abstract to accompany their article when it is published. These video abstracts, known as Science Sketches, are up to 2 minutes long and will be published on YouTube and then embedded in the article abstract. Science Sketch Editors on the MBoC Editorial Board will provide guidance as you prepare your video. Information about how to prepare and submit a video abstract is available at www.molbiolcell.org/science-sketches. Please contact mboc@ascb.org if you are interested in creating a Science Sketch.

Thank you for submitting your manuscript to Molecular Biology of the Cell. Please do not hesitate to contact this office if you

have any questions.

Sincerely,

Eric Baker
Journal Production Manager
MBoC Editorial Office
mbc@ascb.org

Reviewer #1 (Remarks to the Author):

This short study focuses on the ability of CLASP proteins to connect actin and microtubule networks. Through a series of co-sedimentation and TIRF experiments, the authors show that the interaction of CLASP2 with the two polymers allows the progressive recruitment of actin filaments to the side of single microtubules. They identify a short domain of CLASP that is critical for this function, and the importance of CLASP2 for the organization of the actin cytoskeleton is assessed by a depletion of the protein in smooth muscle cells, which leads to a disorganization of stress fibers.

Comments:

The interactions of CLASP with actin and microtubules have already been reported by other studies, as well as their importance for the organization of the cytoskeleton. The novelty of this study lies in imaging the effect of CLASP on the co-organization of actin and microtubule networks. On this point, quantification of the number of actin filaments recruited is complicated and unconvincing. Countless papers show that it is possible to quantify the fluorescence signal of single actin filaments labeled with fluorescent phalloidin. I don't understand why the authors don't rely here on such a simple calibration for this quantification.

The concentration of F-actin used in these experiments seems very high (6.5 uM) and I imagine that a very large majority of the actin filaments are not visible because they diffuse rapidly in solution. Is such a high concentration of F-actin necessary or not to have a colocalization of F-actin on microtubules? If yes, is it because CLASP has a very low affinity for filamentous actin? If the affinity of CLASP for F-actin is so low, is it reasonable to conclude from these experiments that CLASP could recruit F-actin to microtubules efficiently in cells?

The end of the study on the effect of CLASP depletion on the organization of the actin cytoskeleton seems out of context in this study. While this experiment may indeed show an effect of CLASP on the organization of the actin cytoskeleton, there is no evidence in this experiment (and contrary to what the authors claim) that microtubules would be involved in this interaction.

Reviewer #2 (Remarks to the Author):

The manuscript by Rodgers et al entitled "CLASP2 facilitates dynamic actin filament organization along the microtubule lattice" explores the role of CLASP2 as an actin-microtubule crosslinking protein. The experiments performed and overall conclusions of this study are sound and in-line with previous work exploring the roles of CLASP2 with microtubules and as an actin-microtubule crosstalk protein. The advance of this work lies with the detailed analyses performed and pelleting assays to tease out some direct role for CLASP2 with actin filaments (it does not bundle actin filaments). Overall, the paper is a very nice investigation.

Major comments:
• An earlier, more clear description of the mini-CLASP fragment will help future readers. I found that I missed the value of this construct on the first pass of this work until the discussion section.
• Consider moving the pelleting assays showing that actin-microtubules co-pellet in the presence of CLASP2 to the main figure. This data was very nice and I think helps illustrate the authors' point.
• I like the use of inverted LUTs to show microtubule or actin panels, but sometimes it was difficult to identify which cytoskeleton I was looking at. For Figure 2, recoloring individual panels to different colors and presenting as a time-series Z-stack projection might further emphasize the point that more actin is accumulating/landing on microtubules over the time period. This could make for an easier comparison between CLASP and no CLASP reactions.
• I appreciate the authors used two different siRNAs to presumably knock down CLASP2 and the phenotype with actin looks robust. Can the authors estimate the level of knock down for this phenotype beyond "minimal protein levels were detected 72 hours after transfection". Alternatively, does a "rescue" plasmid restore actin morphology? Also, since this work revolves around actin and microtubule crosstalk, I am curious what the microtubules look like in these cells or if a parameter of linkage changes (i.e., are there fewer instances of actin-microtubule overlap in CLASP2 siRNA cells).

Minor comments and curiosities beyond the scope of this current work:
• Does CLASP2 alter actin dynamics? (i.e., the number of actin filaments present or their elongation rates in the absence of microtubules?)
• Do bundling activities change in the presence of dynamic parameters in assays similar to Prezel et al., 2017 (Methods in Cell Biology).
• The actin "bridges" formed are pretty neat. Does the dynamic instability of microtubules unlink actin and microtubule activities?
• I was surprised that phalloidin-stained actin did not alter CLASP2 binding. The authors mention this in the text, but should probably state this with the first use of phalloidin-actin for clarity.
• Does the second Tog domain mediate these interactions on its own? Are the effects linking actin-microtubules the same when CLASP2 Tog2 is replaced with similar or divergent XMAP Togs or vice versa?
• What is the biological significance of the 11 actin filaments per bundle? Related, what happens if the authors make preformed actin filament bundles with Fascin (makes 30 filament bundles) or another bundler to assess whether bundle dimensions are important for CLASP2's "actin bundle to microtubule" linking activity.
• The experiments with EB proteins mentioned in the discussion sound exciting.

Rodgers et al. "CLASP2 facilitates dynamic actin filament organization along the microtubule lattice"

Response to Reviewers

We thank the reviewers for their valuable and constructive feedback. We have now addressed all points raised by the reviewers and requested by the editor. In brief, we have extensively expanded our investigation of the effects of CLASPs on the actin network in cells. Specifically, (i) we include a Western Blot demonstrating CLASP siRNA depletion levels (new Supplemental Figure S2), (ii) we have performed rescue experiments, which demonstrate that actin organization is restored in CLASP2α rescue conditions (revised Figure 4), (iii) we now include representative images of both microtubule and actin networks in non-targeting and CLASP depleted cells, which demonstrate increased misalignment between microtubules and actin filaments in CLASP-depleted conditions (revised Figure 4). Furthermore, we have determined the binding affinity of the L-TOG2-S minimal CLASP2 construct to actin filaments to be 1.1 ± 0.6 μM (revised Figure 1, panel 1E). Finally, we clarify our motivation for performing the more complex 'stepping' analysis, and show that it provides a more accurate investigation of actin landing events, when compared to a simpler, intensity-calibration-based method (please see the response to Reviewer 1 below).

Notably, in the process of determining CLASP-actin binding affinity, we found that the buffer conditions previously used by *Engel et al., Cytoskeleton 2014* in their CLASP-actin co-sedimentation assay cause protein aggregation in our assays. We have thus decided to opt for a more direct, microscopy-based approach to investigate CLASP's ability to bundle F-actin filaments (revised Supplemental Figure 1), rather than using a bulk co-sedimentation method. Importantly, this has not changed any of our previous conclusions, as we find that CLASP does not bundle F-actin filaments in the conditions used in our assays.

Overall, we believe that these additional results and analyses have significantly strengthened our manuscript.

Below, we elaborate on all of the revisions made and provide a point-by-point response to reviewers' comments.

Reviewer #1 (Remarks to the Author):

This short study focuses on the ability of CLASP proteins to connect actin and microtubule networks. Through a series of co-sedimentation and TIRF experiments, the authors show that the interaction of CLASP2 with the two polymers allows the progressive recruitment of actin filaments to the side of single microtubules. They identify a short domain of CLASP that is critical for this function, and the importance of CLASP2 for the organization of the actin cytoskeleton is assessed by a depletion of the protein in smooth muscle cells, which leads to a disorganization of stress fibers.

Comments:
The interactions of CLASP with actin and microtubules have already been reported by other studies, as well as their importance for the organization of the cytoskeleton. The novelty of this study lies in imaging the effect of CLASP on the co-organization of actin and microtubule networks. On this point, quantification of the number of actin filaments recruited is complicated and unconvincing. Countless papers show that it is possible to quantify the fluorescence signal of single actin filaments labeled with fluorescent phalloidin. I don't understand why the authors don't rely here on such a simple calibration for this quantification.

We are aware of previous reports using fluorescence intensity measurements to estimate the number of actin filaments in an actin bundle (e.g. Breitsprecher et al., *J Cell Sci* 2011 and Park et al., *FEBS Lett* 2020). In our experiments, we found that simply measuring the fluorescence intensity of the actin filaments in microtubule regions was not a reliable approach.

Specifically:

- We find a significant variability in the illumination across our field of view, which is not uncommon for the TIRF imaging approach. Using microtubules as our 'standard candle' because of their distinguished morphology and brightness, we determined that the microtubule intensity can range between 3300 – 7500 au, depending on their location in the TIRF field (please see Rebuttal Figure 1A below). The individual standard deviations are also large (700 – 1000 au), meaning that there is additional significant variability along the microtubule lattice. Such large variability can cause uncertainty in determining the absolute intensity of a single filament even for microtubules, let alone for single F-actin filaments.
- In addition, we find that the background noise in our images is increasing significantly over time (Rebuttal Figure 1B). This is not surprising, given that more and more actin particles are being retained at the surface in our experiments over time. Notably, neither of the above references specifically addressed how the background noise is accounted for or how it may impact the determination and validity of a single-filament brightness 'standard' over time.
- Finally, using kymograph analysis and an established automated stepping algorithm (previously published by *Bronson et al., Biophys J 2009*) in lieu of a manual intensity analysis allowed us to be completely agnostic with respect to the size of the particles that land (i.e. whether we are observing landing of single actin filaments versus bundles). In our manuscript, we strictly reported the number of landing events, rather than the absolute number of actin filaments on microtubule regions. Nevertheless, based on our results demonstrating that CLASP2α on its own does not bundle F-actin in these conditions, we conclude that most of the observed steps are indeed due to landing of single actin filaments.

Additionally, we directly compared a manual analysis approach of estimating F-actin bundle size against our kymograph-stepping analysis (Rebuttal Figure 1C-D). We took a closer look at our representative example from Figure 2C of the manuscript for this comparison. We first manually determined the single F-actin intensity by producing an intensity line scan for the first F-actin landing event (Rebuttal Figure 1C). Then, we estimated the background intensity by averaging the intensity along the microtubule lattice. For the same line scan, we then measured the F-actin signal intensity in the last frame. Dividing the last frame intensity by the single F-actin intensity, we determined a total number of six F-actin on the microtubule. In contrast, our stepping analysis showed a total of five F-actin landing events (Rebuttal Figure 1D). This was due to some variability in the intensity of the F-actin landing events (Rebuttal Figure 1D). While the 'step size' of the individual events was variable, the largest step had an intensity of 8500 AU, which we think still corresponds to a single F-actin filament. Overall, we think that our analysis more adequately represents the properties of F-actin accumulation on CLASP-coated microtubule lattice regions than a simpler calibration approach.

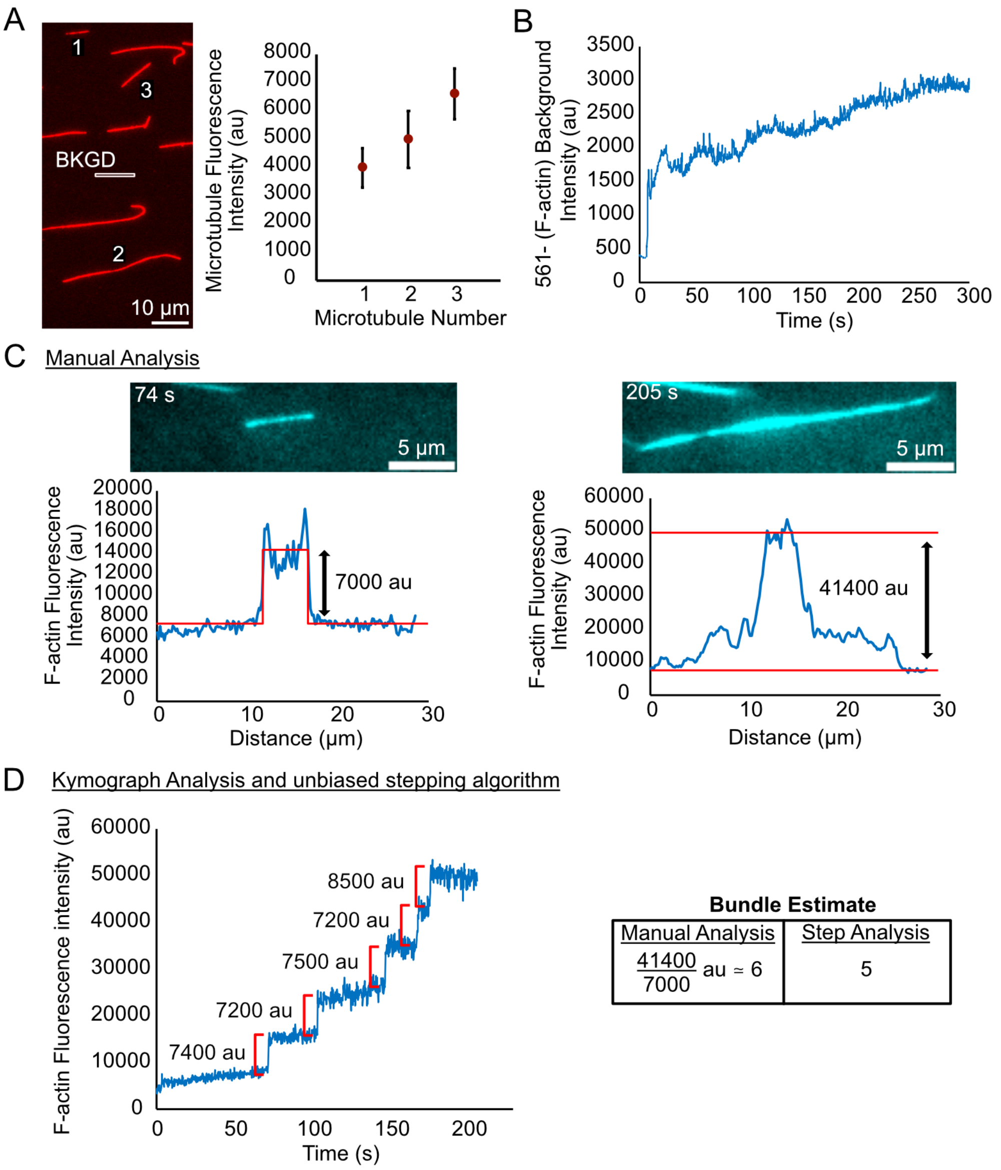

Rebuttal Figure 1. Kymograph and stepping analysis improve the estimation of the number of F-actin landing events onto CLASP2α-coated microtubules. *A) Left: a TIRF image of microtubules corresponding to experiment used for Figure 2. Right: the mean fluorescence intensities of the microtubules denoted by corresponding numbers on the left, with standard deviation. B) The background intensity, as measured in the white box in panel B, for the 561-nm (F-actin) channel over time. The initial jump in intensity corresponds to introduction of F-actin into the channel. C) Example of a manual analysis used to estimate the number of F-actin based on intensity calibration. Left, the first F-actin landing event at 74 seconds. Right, the last frame of the analysis at 205 seconds. The two images are scaled the same. Plots of the F-actin fluorescence intensity versus distance along the line scans. Blue line is the raw intensity, and the red line is a representation of the background intensity and average intensity measurements. D) Plot of the F-actin fluorescence intensity over time for a 3-pixel line scan from the kymograph in Figure 2C. Blue line is the raw intensity. Red brackets represent stepping analysis estimation for F-actin step size. Step fit is shown in Figure 2C of the manuscript.*

The concentration of F-actin used in these experiments seems very high (6.5 uM) and I imagine that a very large majority of the actin filaments are not visible because they diffuse rapidly in solution. Is such a high concentration of F-actin necessary or not to have a colocalization of F-actin on microtubules? If yes, is it because CLASP has a very low affinity for filamentous actin? If the affinity of CLASP for F-actin is so low, is it reasonable to conclude from these experiments that CLASP could recruit F-actin to microtubules efficiently in cells?

The reviewer raises an excellent point. Indeed, high concentrations of F-actin are not necessary for this interaction. We have performed experiments testing lower concentrations of F-actin and observed CLASP-dependent crosslinking of actin filaments to microtubules at concentrations as low as 560 nM F-actin, as shown in the Rebuttal Figure 2 below.

560 nM F-actin + 100 nM CLASP2α-GFP after 30 minutes

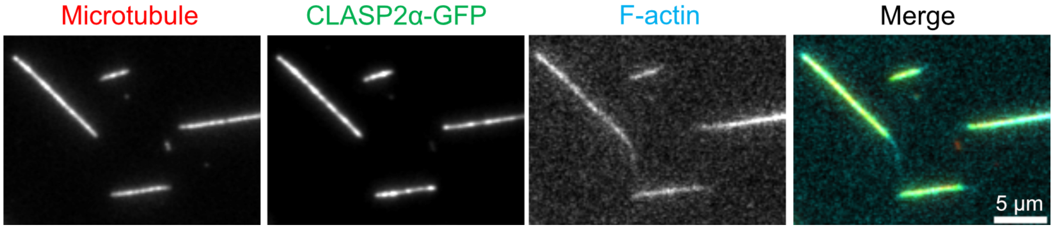

Rebuttal Figure 2. Lower concentrations of F-actin also co-localize with CLASP2α-coated microtubules. *Example image of 560 nM TRITC-phalloidin F-actin colocalizing with Alexa 647-labeled taxol microtubules and 100 nM CLASP2α-GFP after 30 minutes. Experiment done in duplicate.*

Our reasoning behind using a higher concentration of F-actin (6.5 µM) in our manuscript was that in these higher-concentration conditions we observed a clear saturation in the actin signal on microtubules within a few minutes, which was well within the duration of the experiments (main Figure 2B). Since we were interested in investigating the total amount of actin filaments that can be supported by CLASP2 on a microtubule, we wanted to make sure that we are reaching saturation in our experiments. Notably, the concentrations of actin in non-muscle cells are estimated to be an order of magnitude greater (~100 µM, *Pollard et al. Ann Rev Biophys Biomol Struct, 2000*), thus, the concentrations used in our assay are well below physiological concentrations.

In addition, prompted by the reviewer's and editor's comments, we have now performed binding affinity measurements for the interaction between the minimal L-TOG2-S CLASP construct and F-actin. We found that the interaction between L-TOG2-S and F-actin in the absence of microtubules is indeed quite weak, with the affinity of 1.1 ± 0.6 µM (revised Figure 1E). Given such a low-affinity interaction, the amounts of purified protein necessary for the conventional pelleting assays did not allow us to determine the affinity of full-length CLASP2α binding to F-actin. Namely, unlike the minimal CLASP construct, full length CLASP2α cannot be bacterially-expressed, and the yields from our Sf9-insect-cell-based expressions are typically significantly lower. Nevertheless, we think that the determined binding affinity of the minimal construct provides a reasonable order-of-magnitude estimate of the strength of the CLASP-F-actin interaction in the absence of microtubules for the following reasons:
(i) In our new investigation of whether full length CLASP2 is able to bundle F-actin filaments on its own, we did not observe significant co-localization of 200 nM CLASP2α with 1 µM F-actin (please see Rebuttal Figure 3A below), suggesting a weak direct interaction between CLASP2 and F-actin, consistent with our L-TOG2-S binding affinity measurement.
(ii) We observe similar total amounts of F-actin landing on CLASP-coated vs. TOG2-coated microtubules, suggesting similar affinities of F-actin binding to either the TOG2, or the full-length protein (main Figure 1, please see quantification in the Rebuttal Figure 3B below).

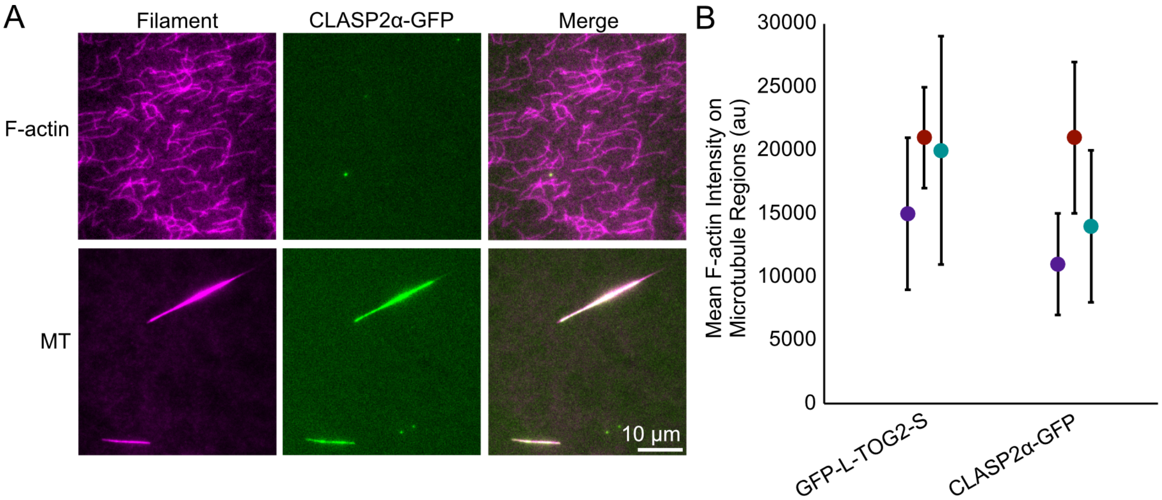

Rebuttal Figure 3. CLASP2α does not strongly co-localize with single actin filaments and the amount of F-actin accumulation is similar between the full length and minimal CLASP2 constructs. *A) Example TIRF microscopy images of F-actin and microtubules (magenta), 200 nM CLASP2α-GFP (green), and merged image after10 minute incubation. CLASP2α images are scaled the same. B) Quantification of the mean F-actin intensity in microtubule regions for experiments performed in the main Figure 1, comparing GFP-L-TOG2-S and CLASP2α-GFP F-actin accumulation after 10-minute incubation. Error bars are the standard deviation and colors correspond to each experimental trial.*

Overall, our results suggest that concentrating CLASP2 on the microtubule is the precursor for F-actin accumulation along the microtubule lattice. Notably, the binding affinity of CLASP for microtubules is much higher (estimated to be 150-200 nM by *Patel et al. Cytoskeleton, 2012*). Thus, although the affinity of CLASP-F-actin interaction is weak on its own, microtubules serve as a platform for this interaction by locally concentrating CLASP along the microtubule lattice, providing a multitude of binding sites for the weak CLASP-F-actin interaction. This can explain why we observe robust actin binding along the microtubule lattice even in the presence of as little as 560 nM CLASP. We speculate that the mechanism whereby locally-increased concentration of CLASP on the microtubule lattice facilitates robust microtubule-CLASP-actin interactions is also relevant in the physiological context.

The end of the study on the effect of CLASP depletion on the organization of the actin cytoskeleton seems out of context in this study. While this experiment may indeed show an effect of CLASP on the organization of the actin cytoskeleton, there is no evidence in this experiment (and contrary to what the authors claim) that microtubules would be involved in this interaction.

We appreciate the reviewer's remark that our data in cells does not show direct evidence for the role of microtubules in the CLASP-F-actin interaction. To that end, we have edited the main text to be more speculative about the mechanism. Furthermore, we have significantly expanded our investigation to more directly assess the potential contribution of CLASP on the microtubule-actin network coordination in vascular smooth muscle cells. Specifically, we now include images of both microtubule and actin networks in non-targeting and CLASP depleted cells, which demonstrate loss of actin-microtubule co-alignment when CLASPs are depleted (revised Figure 4E-G). Combined with the CLASP rescue experiments in cells (revised Figure 4D, please see response to Reviewer 2 below), our new results strengthen our speculation that the microtubule-CLASP2-actin interaction may be involved in the formation of higher-order actin structures in cells.

Reviewer #2 (Remarks to the Author):

The manuscript by Rodgers et al entitled "CLASP2 facilitates dynamic actin filament organization along the microtubule lattice" explores the role of CLASP2 as an actin-microtubule crosslinking protein. The experiments performed and overall conclusions of this study are sound and in-line with previous work exploring the roles of CLASP2 with microtubules and as an actin-microtubule crosstalk protein. The advance of this work lies with the detailed analyses performed and pelleting assays to tease out some direct role for CLASP2 with actin filaments (it does not bundle actin filaments). Overall, the paper is a very nice investigation.

Major comments:

• An earlier, more clear description of the mini-CLASP fragment will help future readers. I found that I missed the value of this construct on the first pass of this work until the discussion section.

We thank the reviewer for this point. We have now included a brief description of the minimal CLASP construct earlier in the Results section to help clarify our findings (lines 121 – 124).

• Consider moving the pelleting assays showing that actin-microtubules co- pellet in the presence of CLASP2 to the main figure. This data was very nice and I think helps illustrate the authors' point.

We appreciate the thoughtful comment regarding our co-pelleting result, previously included in the supplement. That result was a replicate of the CLASP-F-actin co-pelleting assay previously published by Engel et al., *Cytoskeleton* 2014 (*Figure 6A*); we thus felt it belonged in a supplemental figure. However, while pursuing further co-pelleting assays to determine the binding affinity of the CLASP-F-actin interaction, we found that CLASP2α aggregated in these buffer conditions (a buffer containing 25 mM HEPES pH 7.25, 100 mM NaCl, and 10 mM $MgCl_2$). We thus opted to perform the full titration co-pelleting assay in more standard F-actin buffer conditions (General Actin Buffer containing 2 mM Tris-HCl pH 8.0, 0.2 mM ATP, 0.5 mM DTT, and 0.2 mM $CaCl_2$, based on Pardee & Spudich *Meth Cell* Biol 1982 and Cytoskeleton, Inc *Actin Binding Protein Biochem Kit Manual v.9.2*). Since these co-pelleting experiments require large amounts of purified protein, we were not able to determine the affinity of the full length CLASP2α (which cannot be bacterially expressed) but have determined the affinity for the minimal L-TOG2-S construct binding to F-actin. As suggested by the reviewer, we now include these new co-pelleting results as a part of the revised main Figure 1.

• I like the use of inverted LUTs to show microtubule or actin panels, but sometimes it was difficult to identify which cytoskeleton I was looking at. For Figure 2, recoloring individual panels to different colors and presenting as a time-series Z-stack projection might further emphasize the point that more actin is accumulating/landing on microtubules over the time period. This could make for an easier comparison between CLASP and no CLASP reactions.

We appreciate the reviewer's suggestion. We have now modified the coloring of the actin filament images in Figure 2A and Video 1, representing them as a heat map over time. We think that this coloring better demonstrates F-actin accumulation on microtubules.

• I appreciate the authors used two different siRNAs to presumably knock down CLASP2 and the phenotype with actin looks robust. Can the authors estimate the level of knock down for this phenotype beyond "minimal protein levels were detected 72 hours after transfection". Alternatively, does a "rescue" plasmid restore actin morphology? Also, since this work revolves around actin and microtubule crosstalk, I am curious what the microtubules look like in these cells or if a parameter of linkage changes (i.e., are there fewer instances of actin-microtubule overlap in CLASP2 siRNA cells).

To address the reviewer's concerns regarding the estimated level of knock down for phenotypes demonstrated in Figure 4, we have now expanded the Supplemental Figure 2 to include: (i) images showing panCLASP antibody staining for both non-targeted control as well as the two siRNA conditions (Supplemental Figure S2A-C); (ii) a representative Western blot demonstrating the CLASP depletion levels (Supplemental Figure 2D).

Furthermore, to address the question if CLASP rescue restores the actin organization phenotype, we have performed CLASP rescue experiments, as suggested. Our new results, included in Figure 4, demonstrate that CLASP2α rescue does restore ventral stress fiber organization (Figure 4D). In addition, we have also included representative images of the actin and microtubule networks in non-targeting and CLASP depleted cells, which demonstrate increased misalignment between microtubules and actin filaments in CLASP depleted conditions (revised Figure 4). Taken together, we think that our expanded investigation provides significant new support to the relevance of CLASP-mediated actin-microtubule interactions in the context of rat vascular smooth muscle cells.

Minor comments and curiosities beyond the scope of this current work:

• Does CLASP2 alter actin dynamics? (i.e., the number of actin filaments present or their elongation rates in the absence of microtubules?)

This is a very interesting question that we are looking forward to pursuing in the future.

• Do bundling activities change in the presence of dynamic parameters in assays similar to Prezel et al., 2017 (Methods in Cell Biology).

We are optimizing co-polymerization experiments with dynamic actin filaments and dynamic microtubules, with the interest of studying the role of CLASP2 in dual dynamic systems. We agree that it would be very interesting to compare actin accumulation on dynamic microtubules, where the specific localization of CLASP2 on the microtubule (e.g. end vs lattice) could potentially play additional roles in these interactions.

• The actin "bridges" formed are pretty neat. Does the dynamic instability of microtubules unlink actin and microtubule activities?

That is another great question which we are looking forward to exploring in the future. Given that CLASP2 is a microtubule stabilizing protein, which suppresses microtubule catastrophe and promotes microtubule rescue (*reviewed in Lawrence et al. JCS 2020*), we expect that microtubules would not be highly dynamic in the presence of CLASP2. Rather, we expect that microtubules would continue to grow and to form larger cytoskeletal tracks on which actin structures can be templated. It is also possible that dynamic microtubules in the presence of CLASP could transport growing actin filaments, as has been observed for the CLIP-170, formin, and EB protein system (Henty-Ridilla et al., *Science* 2016), as well as Gas2L (Willige et al., *EMBO Reports* 2019), which link the two cytoskeletons. We are excited to pursue future investigations along these directions.

• I was surprised that phalloidin-stained actin did not alter CLASP2 binding. The authors mention this in the text but should probably state this with the first use of phalloidin-actin for clarity.

Thank you for pointing this out. We have now included a reference to the supplemental figure in the Figure 1 results when we first use phalloidin (lines 114 – 117).

• Does the second Tog domain mediate these interactions on its own? Are the effects linking actin-

microtubules the same when CLASP2 Tog2 is replaced with similar or divergent XMAP Togs or vice versa?

While we have not performed these experiments, we agree that it would be of great interest to test the role of individual TOG domains in the actin-microtubule crosslinking.

• *What is the biological significance of the 11 actin filaments per bundle? Related, what happens if the authors make preformed actin filament bundles with Fascin (makes 30 filament bundles) or another bundler to assess whether bundle dimensions are important for CLASP2's "actin bundle to microtubule" linking activity.*

This is a great point; 11 actin filaments within a bundle is a relatively small number for cellular actin bundles – e.g. stress fiber bundles are typically composed of up to 30 actin filaments (*Cramer et al. JCB 1997*). However, in this context, we think of the microtubule serving as a template for potential bundle formation by actin bundling proteins, since we have not detected any direct actin bundling activity of CLASP (revised Supplemental Figure 1). An exciting future direction will be to perform experiments with additional actin bundling proteins in solution and determine the potential role of microtubule-templated F-actin accumulation in actin bundle formation.

• *The experiments with EB proteins mentioned in the discussion sound exciting.*

We thank the reviewer for their enthusiasm of our discussion of future experiments.

RE: Manuscript #E22-05-0149R
TITLE: "CLASP2 facilitates dynamic actin filament organization along the microtubule lattice"

Dear Marija

I am pleased to accept your very interesting manuscript for publication in Molecular Biology of the Cell.

Sincerely,
Laurent Blanchoin
Monitoring Editor
Molecular Biology of the Cell

Dear Dr. Zanic:

Congratulations on the acceptance of your manuscript.

A PDF of your manuscript will be published on MBoC in Press, an early release version of the journal, within 10 days. The date your manuscript appears at www.molbiolcell.org/toc/mboc/0/0 is the official publication date. Your manuscript will also be scheduled for publication in the next available issue of MBoC.

Within approximately four weeks you will receive a PDF page proof of your article.

Would you like to see an image related to your accepted manuscript on the cover of MBoC? Please contact the MBoC Editorial Office at mboc@ascb.org to learn how to submit an image.

Authors of Articles and Brief Communications are encouraged to create a short video abstract to accompany their article when it is published. These video abstracts, known as Science Sketches, are up to 2 minutes long and will be published on YouTube and then embedded in the article abstract. Science Sketch Editors on the MBoC Editorial Board will provide guidance as you prepare your video. Information about how to prepare and submit a video abstract is available at www.molbiolcell.org/science-sketches. Please contact mboc@ascb.org if you are interested in creating a Science Sketch.

We are pleased that you chose to publish your work in MBoC.

Sincerely,

Eric Baker
Journal Production Manager
MBoC Editorial Office
mbc@ascb.org

Reviewer #2 (Remarks to the Author):

I like this revised version of the manuscript. The authors have gone above and beyond to alleviate my previous concerns.

I really appreciate the way the authors incorporated cell analyses for actin and microtubules in the revised version. Those particular cells are really striking.
